# Landscape of homologous recombination deficiencies in solid tumours: analyses of two independent genomic datasets

**DOI:** 10.1186/s12885-021-09082-y

**Published:** 2022-01-03

**Authors:** Zhongwu Lai, Matthew Brosnan, Ethan S. Sokol, Mingchao Xie, Jonathan R. Dry, Elizabeth A. Harrington, J. Carl Barrett, Darren Hodgson

**Affiliations:** 1 AstraZeneca, Waltham, MA 02451 USA; 2 Foundation Medicine Inc., Cambridge, MA USA; 3 Present Address: Tempus Labs Inc., Boston, MA USA; 4 AstraZeneca, Cambridge, UK

**Keywords:** Homologous recombination deficiency, Homologous recombination repair, Genomic loss of heterozygosity, Loss of function, cancer, Breast, Ovarian, Germline, Somatic, PARP inhibitors, Immune checkpoint inhibitors

## Abstract

**Background:**

DNA repair deficiencies are characteristic of cancer and homologous recombination deficiency (HRD) is the most common. HRD sensitizes tumour cells to PARP inhibitors so it is important to understand the landscape of HRD across different solid tumour types.

**Methods:**

Germline and somatic BRCA mutations in breast and ovarian cancers were evaluated using sequencing data from The Cancer Genome Atlas (TCGA) database. Secondly, a larger independent genomic dataset was analysed to validate the TCGA results and determine the frequency of germline and somatic mutations across 15 different candidate homologous recombination repair (HRR) genes, and their relationship with the genetic events of bi-allelic loss, loss of heterozygosity (LOH) and tumour mutation burden (TMB).

**Results:**

Approximately one-third of breast and ovarian cancer BRCA mutations were somatic. These showed a similar degree of bi-allelic loss and clinical outcomes to germline mutations, identifying potentially 50% more patients that may benefit from precision treatments. HRR mutations were present in sizable proportions in all tumour types analysed and were associated with high TMB and LOH scores. We also identified numerous BRCA reversion mutations across all tumour types.

**Conclusions:**

Our results will facilitate future research into the efficacy of precision oncology treatments, including PARP and immune checkpoint inhibitors.

**Supplementary Information:**

The online version contains supplementary material available at 10.1186/s12885-021-09082-y.

## Background

The development of precision anti-cancer medicines requires the exploitation of a tumour-specific genetic or biological alteration. Deficiencies in DNA damage response (DDR) mechanisms, which are a hallmark of cancer, are an appropriate area to target.

Different types of DNA damage are repaired by different DDR mechanisms and homologous recombination repair (HRR) is the high-quality pathway for repairing DNA double-strand breaks (DSBs). Deleterious mutations in HRR pathway genes, of which *BRCA1* and *BRCA2* are the most characterized, can lead to a deficiency in repair by homologous recombination in tumour cells that is exploited in poly (ADP-ribose) polymerase (PARP) inhibitor treatment [1–4].

Tumour suppressor genes, including *TP53*, *BRCA1* and *BRCA2*, typically lose their functionality through bi-allelic loss of function (LoF), primarily caused by a loss of heterozygosity (LOH) resulting from a mutation in one allele and secondary loss of the remaining wild-type allele. It can also occur from copy number neutral LOH (two alleles but with same identical mutation), or a compound heterozygous mutation (two different mutations at each allele of a particular gene locus). Mutations can either be germline (inherited) or somatic (acquired) and initial clinical trials of PARP inhibitors were conducted in ovarian cancer patients with germline BRCA mutations; however, subsequent studies suggested that somatic mutations in HRR genes, including *BRCA1* and *BRCA2*, had a similar biological phenotype to germline mutated tumours and these patients had also been shown to benefit from treatment with PARP inhibitors [5–7].

To quantify DNA repair deficiency, measurements such as detection of mutations in HRR genes including *BRCA1* and *BRCA2*, the homologous recombination deficiency (HRD) score [8], percent genome wide-LOH [9], tumour mutation burden (TMB) and microsatellite instability (MSI) are increasingly being used to investigate the cause (e.g., a gene mutation) and effect (e.g., HRD score) of deficiencies in DNA repair processes [10–12]. The HRD score is the sum of three independent DNA-based measurements of genomic instability, namely telomeric allelic imbalance (TAI), large-scale transitions (LST) and LOH [8]. TMB is a quantitative measure of the total number of somatic mutations per region sequenced of a tumour genome and is an emerging biomarker for response to immunotherapy [13–15]. MSI is a characteristic of a defective DNA mismatch repair process and tumours with high MSI levels are more susceptible to immune-enhancing therapies; in 2017 the FDA granted accelerated approval of pembrolizumab [16], a programmed cell death 1 (PD-1) inhibitor, for patients whose cancers have high MSI regardless of tumour type.

Measurements of DNA repair deficiencies are useful in the design of clinical trials of precision treatments that target deficiencies in the DNA repair pathways as they can help predict responses to established or newly developed anti-cancer therapies such as DNA damaging chemotherapy, PARP inhibition and immunotherapy approaches. Therefore, to further understand the HRD landscape, including prevalence, co-occurrence and penetrance of the HRD gene phenotype, our study aimed first to determine from an independent genomic dataset of ovarian and breast cancer tumours the frequency of germline and somatic mutations in *BRCA1* and *BRCA2* (BRCAm) and to quantify the degree of bi-allelic LoF and relationship with clinical outcomes of progression-free survival (PFS) and overall survival (OS). Secondly, we expanded our investigation and analysed a much larger dataset of multiple solid tumour types with the aim of verifying our findings from part one and additionally to determine the frequency of germline and somatic mutations across a panel of 15 different HRR genes and their relationship with other DNA repair deficiency measurements.

## Methods

For the first part of our study we re-analysed sequencing data from The Cancer Genome Atlas (TCGA) genomic database using VarDict, a variant caller that is sensitive in detecting insertions and deletions common in tumour suppressors, including *BRCA1* and *BRCA2* [17], to identify germline and somatic BRCA mutations in the TGCA breast and ovarian cancer cohorts (1549 samples in total). Identified mutations were then analysed using mathematical modelling techniques [18] to determine their status of bi-allelic LoF. HRD scores were obtained from Marquard et al [19]. The student t-test was used to examine the relationship between BRCA status, bi-allelic LoF status, and HRD scores and clinical characteristics, including age at diagnosis, and hormone receptor status. Kaplan-Meier survival analysis was used to examine their relationship with PFS and OS.

For the second part of our study, we obtained the sequencing data from a large Foundation Medicine (Foundation Medicine Inc., MA, US) genomic dataset of ~ 75 k anonymized tumour samples, consisting of six major tumour types (bladder, breast, lung, ovarian, pancreatic, and prostate). Samples were required to have at least 20% tumour purity by computational purity assessment and can be successfully analysed by somatic-germline-zygosity (SGZ) algorithm as described in Sun et al [18]. Limited clinical information was available for these samples, but the majority were stage IV disease, as they had sample sites different from the disease type, suggesting metastasized disease. The samples were sequenced at Foundation Medicine Inc. by FoundationOne [20] or FoundationOne CDx assay using the standard panel of at least 324 genes of which deleterious HRR mutations were interrogated in any of the 15 genes in the panel: *ATM, BARD1, BRCA1, BRCA2, BRIP1, CDK12, CHEK1, CHEK2, FANCI (*FoundationOne panel only)*, FANCL, PALB2, RAD15B, RAD51C, RAD51D* and﻿ *RAD54L*. Deleterious mutations detected by the FoundationOne assay are frameshift indels, nonsense, known deleterious missense and splice site mutations, homozygous copy number loss, and large truncating rearrangements. An advanced analytical SGZ algorithm [18] was used to determine germline and somatic BRCAm status, and LOH status, TMB and MSI were determined as described by Chalmers et al^12^ and percentage genome wide-LOH score was based on an algorithm derived from Pawlyn *et al* [21–23]. Trans- and cis-mutations were manually inspected using an Integrative Genomics Viewer (IGV) for evidence of secondary BRCA reversion mutations, which is required to be in-cis with the primary mutation.

The bi-allelic loss status for a HRR gene in a sample was categorized into three classes: positive, negative, or unknown. To determine whether a gene has bi-allelic loss, the following rules were applied in the order of:

1. A sample will be classified as bi-allelic loss positive for the gene if any of the following conditions are met for the given gene:
  - A deleterious mutation of the gene is classified as homozygous by SGZ algorithm, regardless germline or somatic.
  - A gene has two or more deleterious mutations and is considered as composite heterozygous, which will lead to biallelic loss without LOH
  - A homozygous deletion of the gene is reported in the sample.
2. A sample will be classified as bi-allelic loss negative if both of the following conditions are met:
  - No deleterious mutations of the gene are classified as bi-allelic loss in the first step.
  - There is only one mutation of the gene reported and is classified as “het” by SGZ algorithm.
3. A sample will also be classified as bi-allelic loss negative if the mutation of the gene it carries is classified as “germline not_in_tumour”, in which the tumour lost the germline mutant copy, suggesting the mutation isn’t present in the tumour.
4. Otherwise, a sample will be classified as bi-allelic loss unknown. This includes samples where the deleterious mutation is a rearrangement as there is no SGZ prediction available for this variant type.

## Results

### Assessment of somatic and germline BRCA mutations and bi-allelic loss in breast and ovarian cancer

In the first part of our study, raw sequencing data for tumour samples from 467 patients with ovarian cancer (high-grade serous carcinoma) and 1082 with breast cancer (all subtypes) were downloaded and from the TCGA dataset and analysed using VarDict [17]. In total, 14% (219/1549, 95% confidence interval [CI]: 12.5, 16.0) of tumour samples had a deleterious BRCA mutation detected. Among them, 12% (180/1549, 95% CI: 10.1, 13.3%) could be evaluated for germline or somatic status, of which 67% (120/180, 95% CI: 59.2, 73.4) were germline (15% ovarian; 5% breast) and 33% (60/180, 95% CI: 26.6, 40.8) somatic (8% ovarian; 2% breast) (Table 1). Of the total 180 samples with a germline or somatic mutation, 156 (84 ovarian and 72 breast) were available for bi-allelic analysis. Bi-allelic loss of BRCA mutations was seen to be frequent in both germline and somatic mutations, ranging from 81 to 100% for *BRCA1* and 92 to 100% for *BRCA*2 mutations (germline and somatic) for ovarian tumours and 83–96% and 78–80%, respectively in breast tumours (Table 2).

**Table 1:** BRCA mutation status in TCGA ovarian and breast cancer cohorts

Category, *n* (%)	Ovarian (*n* = 467)	Breast (*n* = 1082)
Tumour BRCA mutation	120 (26)	99 (9)
Germline	70 (15)	50 (5)
Somatic	36 (8)	24 (2)
Unknown	14 (3)	25 (2)
Non-BRCA	347 (74)	983 (91)

**Table 2:** Bi-allelic loss of function in the TCGA ovarian and breast cancer cohorts

*n/N* (%)	Ovarian^a^		Breast	
	Germline	Somatic	Germline	Somatic
*BRCA1*	34/34 (100)	13/16 (81)	22/23 (96)^b^	10/12 (83)
*BRCA2*	24/26 (92)	8/8 (100)	21/27 (78)^c^	8/10^d^ (80)
Total	58/60 (97)	21/24 (88)	43/50 (86)	18/22^d^ (82)

*TCGA* The Cancer Genome Atlas

^a^As a result of limitations of access to raw data, only 60 of 70 germline mutations and 24 of 36 somatic mutations in the ovarian cohort were analysed for bi-allelic loss

^b^One patient had one germline and one somatic *BRCA1* mutation assumed to be bi-allelic

^c^One patient had one germline and one somatic *BRCA1* mutation assumed to be bi-allelic; two patients have both germline and homozygous deletions counted as bi-allelic

^d^Two samples with large rearrangements could not be determined for bi-allelic status and were therefore excluded in the bi-allelic calculation

HRD scores had previously been shown as a measure of the degree of HRD in tumour cells [8]. We obtained the HRD sores for these tumours [19] and found that tumours with germline or somatic BRCA mutations have a similar distribution of HRD scores, with germline tumours having slightly higher median scores compared with somatic in both ovarian (69 and 68, respectively, t-test *P* value = 0.48) and breast tumours (75 and 70, respectively, t-test *P* value = 0.35). However, both germline and somatic BRCAm had notably higher median scores than those without a BRCAm (56 and 27, respectively), with a t-test *P* value of < 0.001 and 0.0003 for germline and somatic, respectively in ovarian, and < 0.001 for both germline and somatic, in breast (Supplementary Fig. 1). Patients with germline BRCA mutations were also diagnosed at a younger age than patients with somatic BRCA mutations in both the ovarian and breast cancer cohorts (Supplementary Fig. 2).

In breast cancer, it has been found that triple-negative breast cancer (TNBC) subtype had higher overall mutation rate in BRCA, especially *BRCA1* [24]. We then investigated whether there was difference in bi-allelic loss rate between oestrogen receptor-positive (ER+) and -negative (ER-). No obvious difference was observed in the degree of bi-allelic loss between ER+ and ER- breast cancer tumour subtypes, where 33/37 (89%) ER+ with BRCA mutations, and 29/30 (97%) ER- with BRCA mutations were found to have bi-allelic loss, including two ER- patients with composite heterozygous mutations (one patient had two deleterious mutations in *BRCA1* and one patient had two deleterious mutations in BRCA2) (Supplementary Table 1).

**Fig. 1:**
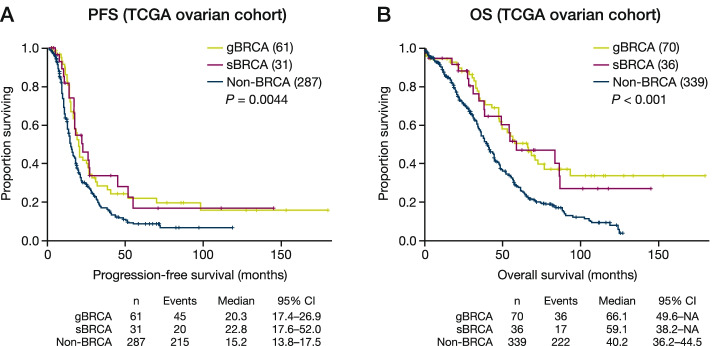
Kaplan-Meier estimates of **a** progression-free survival and **b** overall survival by BRCA status in the TCGA ovarian cancer cohort

With regard to treatment outcomes, ovarian cancer patients with germline or somatic BRCA mutations had similar PFS (median 20.3 months, with 95% CI: 17.4, 26.9 for germline, and 22.8 months with 95% CI: 17.3, 45.2 for somatic, respectively) and OS (median 66.1 months with 95% CI: 48.3, 76.9 for germline, and 54.6 months with 95% CI: 35.2, 87.0 for somatic, respectively) outcomes that were more favourable than those observed in patients without BRCA mutations (PFS median 15.4 months, 95% CI: 13.7, 17.7 and OS median 41.0 months, 95% CI: 36.2, 44.5) (Fig. 1). As platinum therapy is the standard care in ovarian cancer, which targets cancer cells with HRD, such as those with BRCA mutations, it is not surprising that they received greater benefits. The similar benefit between somatic and germline BRCA suggested that somatic BRCA is pheno-copy of germline BRCA. No source data were available for platinum sensitivity in breast cancer treatment to enable a comparative analysis to be conducted for breast cancer patients.

In the second part of our study, tumour samples from 2732 patients with ovarian cancer and 5381 with breast cancer (unknown hormonal subtypes), sourced from the Foundation Medicine genomic dataset were re-analysed. In agreement with the TCGA data, the degree of bi-allelic LoF was high; 96% for *BRCA*1 (both germline and somatic), 89 and 90% for *BRCA*2 (germline and somatic, respectively) in ovarian tumours and 86–91% and 75–86%, respectively, in breast tumours. Again, in agreement with the TCGA data, bi-allelic loss of function was similar across germline and somatic BRCA mutations and across *BRCA1* and *BRCA2* mutations (Table 3).

**Table 3:** Bi-allelic loss of function in the Foundation Medicine ovarian and breast cancer cohorts

*n/N* (%)	Ovarian		Breast	
	Germline	Somatic	Germline	Somatic
*BRCA1* bi-allelic LoF	96/100 (96)	82/85 (96)	77^a^/85 (91)	48^bc^/56 (86^b^)
*BRCA2* bi-allelic LoF	34/38 (89)	47/52 (90)	95^b^/111 (86^b^)	51^bd^/68 (75^b^)

*LoF* loss of function

^a^One tumour lost a germline but gained a homozygous somatic mutation

^b^Composite heterozygous mutations are considered as bi-allelic LoF

^c^The patient with a compound heterozygous LoF had two somatic frameshift mutations

^d^Of the five patients with compound heterozygous LoF, four had both germline and somatic mutations and one had two somatic mutations

**Fig. 2:**
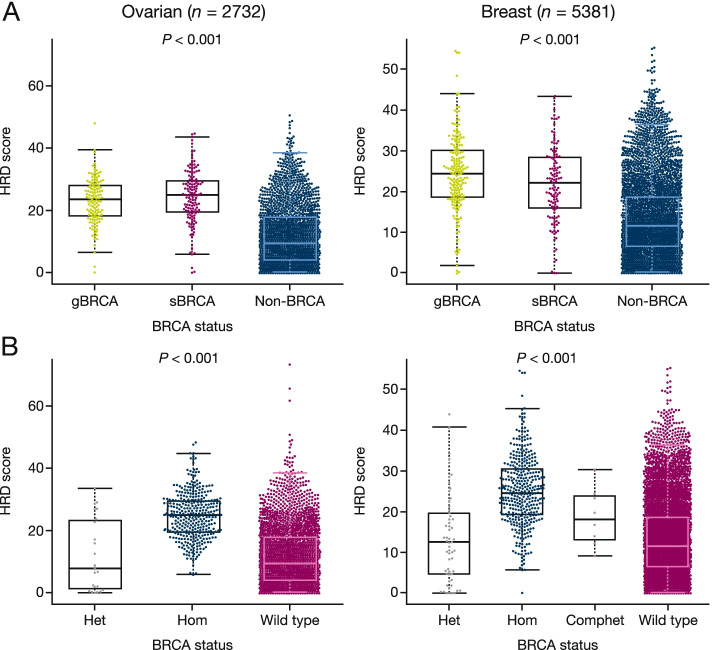
HRD-LOH scores by BRCA mutation in Foundation Medicine ovarian and breast cancer cohorts **a** germline versus somatic versus wild-type, **b** heterozygous (mono-allelic loss without LOH) versus homozygous (bi-allelic loss of function with LOH) versus compound heterozygous (bi-allelic loss of function without LOH) (observed in breast cancer cohort only) versus wild-type. Comphet, compound heterozygous; Het, heterozygous; Hom, homozygous

Agreement between the TCGA and Foundation Medicine datasets was also observed for percent genome wide-LOH scores, which is a measurement of homologous recombination deficiency similar to HRD [8, 9], in that they were similar for germline compared with somatic in both ovarian (23 and 24, respectively) and breast tumours (25 and 23, respectively) and notably higher than the scores for those without a BRCAm (10 and 11, respectively (Fig. 2a). We also showed that bi-allelic LoF was associated with higher percentage genome wide-LOH score: homozygous (bi-allelic LoF with LOH) mutations had scores of 22 and 25 for ovarian and breast cancer, respectively; heterozygous (mono-allelic LoF without LOH) mutations had scores of 8 and 12; while patients without BRCA mutations had scores of 8 and 11, and composite heterozygous (bi-allelic LoF without LOH – breast cancer only) a score of 18 (Fig. 2b). For ovarian cancer, a threshold value of 16 has been established as a clinical cut-off for genome wide-LOH scores in ARIEL3 trial to have clinical utility [23, 25]. However, outside ovarian cancer, there is neither an established cut-off nor validated clinical utility.

When comparing percent genome wide-LOH scores between *BRCA1* and *BRCA2* mutated tumours in both breast and ovarian cohorts, the scores were higher for *BRCA1* than *BRCA2* in ovarian (28 vs. 20, *P* < 0.001), and in breast (25 vs. 22, *P* < 0.001) (Supplementary Fig. 3). Additionally, oncoprint analysis showed that *BRCA1* and *BRCA2* mutations, whether germline or somatic, were mutually exclusive in ovarian cancer and breast cancer (Supplementary Fig. 4).

### Assessment of 15 HRR gene mutations across multiple tumour types

Following assessment of somatic and germline BRCA mutations in the Foundation Medicine ovarian and breast cancer cohorts, the landscape of mutations in a panel of 15 HRR genes was examined in ~ 75,000 clinical samples covering six solid tumour types. Deleterious mutations in any one of these 15 candidate genes, which are potentially causative of HRD, ranged from 12.7% (lung cancer) to 25.5% (prostate cancer) across the tumour types (Supplementary Table 2). The distribution of germline versus somatic mutations of the 15 individual HRR genes were examined across different tumour types and the results are shown in Supplementary Table 7. For *BRCA1* and *BRCA2*, breast and pancreatic cancer showed a ratio of 2:1 for germline versus somatic mutations; in ovarian and prostate cancer the ratio was 1:1, while in bladder and lung cancer, mutations were mostly of somatic origin.

**Fig. 3:**
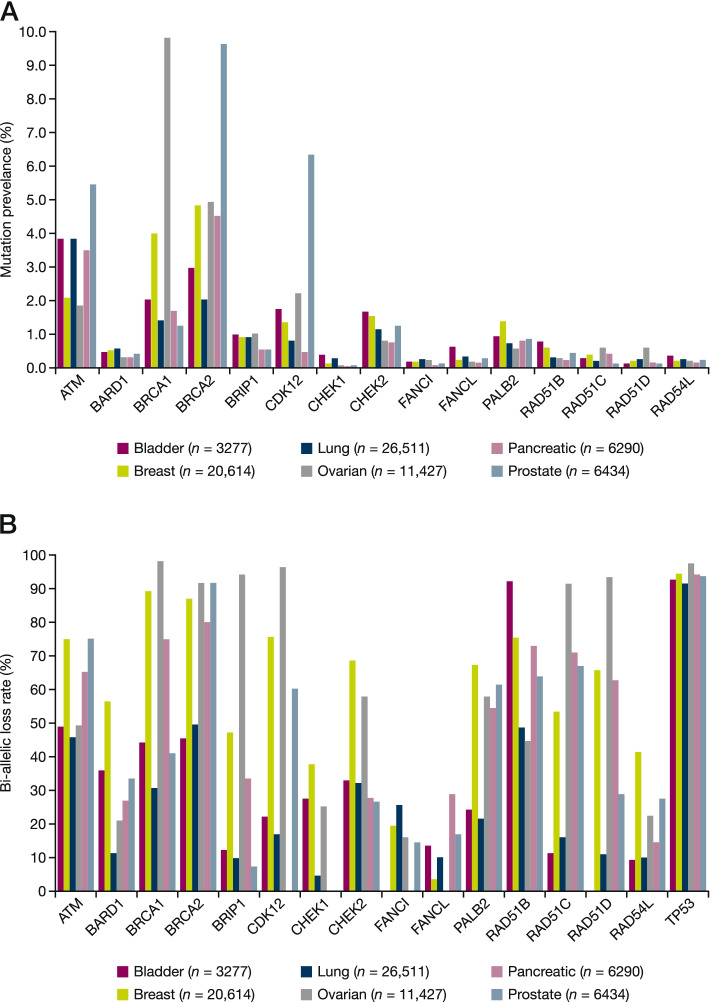
**a** HRR gene mutation prevalence in the Foundation Medicine dataset across six tumour types and **b** bi-allelic loss of function rates of HRR gene mutations in the Foundation Medicine dataset across six tumour types. To prevent multiple counting of a patient so that they are not over-estimated in samples that have a mutation in multiple genes only, one was chosen for representation based upon biological significance, for example, when BRCA is present, it will be called BRCA, even though an *ATM* is also detected

*BRCA1* and *BRCA2* are the most frequently mutated HRR genes, followed by *ATM*. (Fig. 3a). In ovarian cancer, *BRCA1* has 9.8% prevalence, followed by *BRCA2* at 4.9%. Breast cancer has similar *BRCA1* and *BRCA2* prevalence at 4.0 and 4.8%, respectively. Prostate cancer is dominated by *BRCA2* prevalence at 9.6%, while *BRCA1* has only 1.2%. In pancreatic cancer, *BRCA1* and *BRCA2* have a prevalence of 1.7 and 4.5%, respectively. Bladder and lung have a *BRCA1* and *BRCA2* prevalence of between 1.4 and 3.0%. The difference in BRCA rate, as well as preference of *BRCA1* or *BRCA2* in different tissue types suggested that tissue biology might play a role in the difference. *ATM* has significant prevalence between 2 and 5% across tumour types, with 5.4% in prostate. *CDK12* is also highly prevalent in prostate cancer at 6.3%. We also obtained the data for *TP53* (not considered an HRR gene), which showed high mutation rate between 44.3% in prostate and 79.0% in ovarian (Supplementary Table 2).

We next investigated the bi-allelic loss rates for 15 HRR genes among these 6 tumour types. The frequency of bi-allelic loss in HRR genes differed by gene and tumour type and ranged from 34% in lung cancer to 87% in ovarian cancer (Supplementary Table 3). The rate of bi-allelic loss for the 15 HRR candidate genes by tumour type is shown in Fig. 3b, *BRCA2* generally showed high bi-allelic loss rate across tumour types, ranging from 44% in bladder to 90% in ovarian cancer. Ovarian and breast have high bi-allelic loss rates in *BRCA1* at 95 and 91%, respectively, and pancreatic has 67%. *ATM* has bi-allelic loss rates between 40 and 77% among these six tumour types.

Deficiency in DNA repair will lead to more errors in daughter cells, particularly in fast dividing tumour cells. We hypothesized that tumours with HRR mutations will have more homologous recombination errors in DNA, resulting in higher percent genome wide-LOH scores. Higher TMB values were also expected but with less clarity as to whether that would be a cause or an effect of the observed HRR mutation. We thus compared percent genome wide-LOH scores TMB in tumours with mutated HRR (HRRm) versus patients without detectable HRR mutations (HRRwt). Findings showed that HRRm, including BRCA, is significantly associated with higher percent genome wide-LOH and higher TMB scores versus HRR wild-type in the six tumour types examined (Supplementary Table 4; Supplementary Figs. 5 and 6).

It has been shown that only bi-allelic loss of BRCA, but not mono-allelic loss, resulted in elevated HRDetect scores [26]. Indeed, tumours with bi-allelic HRR gene mutations (homozygous or compound heterozygous) also showed higher percent genome wide-LOH scores versus tumours with heterozygous HRR gene mutations (non-LOH) across tumour types (Supplementary Fig. 7).

### HRR mutation and ERBB2 amplification in breast cancer

*BRCA1/2* or HRR mutation prevalence in HER2+ breast cancer remains poorly understood. Though the cohort lacked the clinical information (e.g., fluorescence in-situ hybridization or immunohistochemistry), we sought to use HER2 amplification as the surrogate for HER2+ as it has been shown that HER2 amplification typically results in HER2 overexpression. In the breast cohort, 2024/20,614 (9.8%) were found to have HER2 amplification. In these HER2-amplified samples, 425 (21.0%) had HRR mutation, compared to 3172 (17.1%) out of 18,590 without HER2 amplification (odds ratio [OR] = 1.29 [CI: 1.15, 1.45], Fisher *P* value < 0.0001). Only 101 (5.0%) of 2024 HER2amplified samples carried a BRCA mutation, compared to 1688 (9.1%) in those without HER2 amplification (OR = 0.53 [CI: 0.42, 0.65], Fisher *P* value < 0.0001). This suggests that though HER2-amplified breast cancer has a lower BRCA mutation fraction than HER2-non-amplified breast cancer, it has a higher overall HRR mutation rate.

### Assessment of BRCA reversion mutations in solid tumours

**Fig. 4:**
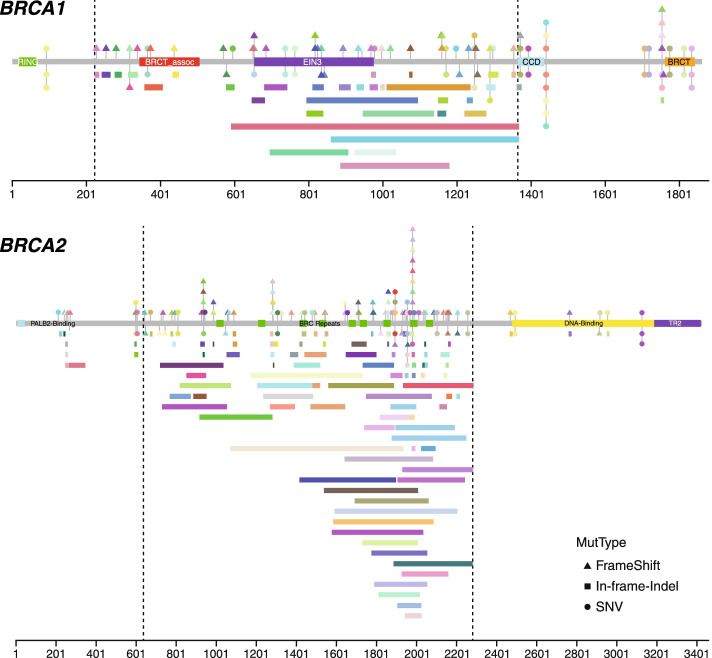
Likely *BRCA1* and *BRCA2* reversion mutations found in the cohort. For each gene, the boxes in the middle indicate protein and domain structures. Numbers below indicate amino acid numbering for the protein. Above the protein structure are the sensitizing mutations and below are candidate reversion mutations. Triangle indicates that the mutation is an indel resulting in frameshift, circle indicates SNV, and rectangle indicates in-frame deletion. Each colour represents a unique sample. The bar below indicates the location of exon 11, the largest exon for *BRCA1* and *BRCA2*

Reversion mutations in BRCA genes, which restored the reading frame of the original frameshift or nonsense mutations, are a known resistance mechanism to platinum-based chemotherapy [27] or PARP inhibitors [28]. In this cohort of anonymized samples, no treatment information was available. Nevertheless, we examined all mutations reported, including those that were predicted to be functionally “unknown”. We found 157 out of 5574 samples with BRCA mutations carried likely BRCA reversion mutations (Fig. 4, Supplementary Table 5) across all six tumour types, ovarian (*n* = 65), breast (*n* = 68), prostate (*n* = 11), pancreatic (*n* = 6), lung (*n* = 5), and bladder (*n* = 2). Of the 157 samples, 56 carried original *BRCA1* mutations, and 101 carried original *BRCA2* mutations.

Of the original sensitizing mutations, 112 are indels (from 1 bp insertion to 11 bp deletion) and 41 are nonsense single nucleotide variations (SNV), two are splice site mutations, and two are missense SNV. In total, 191 putative reversion mutations were identified (Supplementary Table 5), with 17 samples having two reversion mutations, seven having three reversion mutations, and one having four reversion mutations. (Putative reversion mutations are described more fully in the Supplementary section). All large in-frame deletions happened within the largest exon 11 of both *BRCA1* and *BRCA2* (Fig. 4). The largest reversion detection for *BRCA1* is 2650 bp that deletes the 3′ splice site of exon 11, likely resulting known exon 11 alternative splicing isoform, and for *BRCA2* is 2571 bp in exon 11. Both these two large deletions happened in ovarian cancer.

It is also worth noting that the majority of reversion mutations are in exon 11 for both *BRCA1* and *BRCA2*, which is the largest exon for both genes. Thirty-eight (68%) of 55 *BRCA1* cases happened in exon 11, while 86 (85%) of 101 *BRCA2* cases happened in exon 11. The sizes of exon 11 of both *BRCA1* and *BRCA2*, coupled with no critical functional domains, increase the number of mutations that can occur but without significantly impacting the function, resulting in reversion mutations. It has been previously suggested that an alternative splicing isoform resulting in *BRCA1*-Δ11q was responsible for early relapse on PARPi [29]. However, given this data, it might be due to reversion mutations rather than alternative splicing.

We also investigated whether these samples with BRCA reversion mutations might retain the high genomic LOH (gLOH) scores, which are measures of genomic features that should be carried into daughter cells even when new mutations are acquired. In breast and ovarian, where enough reversion samples were identified, we not only observed that gLOH scores remain high in these samples, but also significantly higher than those without a BRCA mutation detected for both breast and ovarian, with t-test *P* values of 6.02e-5 and 7.89e-5, respectively (Supplementary Fig. 8). Of the 157 samples with reversion mutations, 36 (23%) of the sensitizing mutations were predicted to be of germline origin, 58 (37%) of somatic origin, and 63 (40%) of unknown origin. A higher proportion of somatic origin for sensitizing mutations suggest that these tumour cells were highly addictive to the pathway, regardless of the origin.

## Discussion

One of the challenges for personalizing anti-cancer therapies is the identification of predictive biomarkers to select patients who will benefit from the therapy the most. In the first part of our study, we determined from re-analysing data from TCGA that approximately one-third of BRCA mutations in breast and ovarian cancer were somatic (Table 1). Multiple guidelines for BRCA testing exist worldwide for the preventative and therapeutic management of cancer [30, 31]. The identification of patients whose tumours harbour a somatic mutation is of importance because germline testing of blood samples for BRCA mutations does not detect somatic mutations, whereas less commonly performed tumour testing does. Tumour tissue testing will also detect germline BRCAm and a high concordance of results with germline blood testing has been shown [32, 33]. Therefore, because previous studies showed patients with somatic mutations in HRR genes, including *BRCA1* and *BRCA2*, benefited from treatment with PARP inhibitors [5–7], our analysis suggests that tumour testing could identify nearly 50% more patients with BRCA mutations that may benefit from a PARP inhibitor than germline testing alone. When comparing our observed rate of somatic mutations with other published data, the same rate of occurrence of somatic and germline BRCA mutations (one-third vs. two-thirds) was reported, for example, by Winter et al*,* in an unselected population of 273 patients with breast cancer [34]. In high-grade serous ovarian cancer (the most common subtype), 18–30% of all BRCA mutations are reported to be somatic [7], and when broken down into individual genes, rates of 28 and 26% have been reported for *BRCA1* and *BRCA2*, respectively [5, 7, 34].

The number of BRCA mutations we identified with a bi-allelic loss in both the ovarian and breast cancer cohorts was high for both germline and somatic mutations (Table 2). When HRD was quantified by measuring percent genome wide-LOH scores, somatic BRCA mutations showed comparable scores. In addition, somatic BRCA mutations also showed a similar high degree of bi-allelic loss to germline BRCA mutations, and both the somatic and germline BRCA mutations had a higher distribution of LOH scores than patients with no BRCA mutation (Supplementary Fig. 1). This high degree of LOH scores and bi-allelic loss equates to high tumour genomic instability and the similarity in this genetic alteration between the somatic and germline mutations adds to the growing evidence that personalized anti-cancer therapies known to be active against germline mutations may also show clinical activity against somatic BRCA tumours. Of note, the 78% rate of bi-allelic LoF in *BRCA2* in breast cancer we report is considerably higher than the 47% previously reported by Maxwell et al [35].

When hormone status was considered for the breast cancer cohort, no obvious difference was observed in the degree of bi-allelic LoF between oestrogen receptor-positive and -negative tumour subtypes. Differences between patients with breast cancer have been reported before relating to hormone status, for example, *BRCA1* mutations are significantly enriched in TNBC [36]. The clinical outcomes of PFS and OS were similar for patients with ovarian cancer and receiving platinum treatment for both germline and somatic BRCA mutations (Fig. 1). This similarity between the two mutation types again suggests that anti-cancer therapies known to be active against germline BRCA mutations can also show clinical activity against somatic BRCA-mutated tumours. No source data are available to investigate this observation in breast cancer. However, considering the activity of PARP inhibitors previously reported in patients with ovarian cancer harbouring somatic and germline mutations [5, 37, 38], and the similar biology of somatic and germline BRCA mutations in breast cancer we reported, breast cancer patients with somatic mutations may be expected to benefit from PARP inhibitor treatment and a recent case report has indeed shown significant clinical activity of olaparib in a TNBC patient with a somatic *BRCA1* mutation .

In the second part of the study, the findings we describe for the ovarian and breast cancer cohorts in the TCGA dataset were validated and expanded upon using a much larger independent genomic dataset from Foundation Medicine Inc. of nearly 75,000 clinical tumour samples representing six different tumour types. The TCGA data were published and made available first, which is why these data were analysed in the first part of this study, focusing on germline and somatic BRCA mutations in ovarian and breast cancer – two diseases with a known high incidence of BRCA mutations and clinical data supporting the role of PARP inhibitors at the time. Access to the 20 times larger Foundation Medicine cohort was obtained later. The much larger size of the independent cohort made it more appropriate for validation in the second part of the study, and as data were derived from relevant clinical settings and testing (using formalin-fixed, paraffin embedded samples), it ensured that results were clinically relevant.

A similar high bi-allelic loss rate was observed for breast and ovarian cancer in the Foundation Medicine dataset (Table 3) as was observed in the TCGA dataset (Table 2). Furthermore, somatic BRCA mutations again showed a similar high degree of bi-allelic LoF to germline BRCA mutations that was higher for both types of mutation compared with patients with no BRCA mutations (Fig. 2).

When comparing HRD-LOH scores between *BRCA1* and *BRCA2* mutated tumours in both breast and ovarian cohorts (Supplementary Fig. 3), an analysis not performed in the smaller TCGA dataset, the scores were slightly higher for *BRCA1* compared with *BRCA2* for both tumour types. Oncoprint analysis of the Foundation Medicine dataset also showed that *BRCA1* and *BRCA2* mutations, and germline and somatic mutations, were mutually exclusive in ovarian cancer and breast cancer (Supplementary Fig. 4).

HRR gene mutations were found in a notable portion of patients across all six tumour types (HRR mutation rate 13–26%) of which mutations in *TP53* occurred considerably more frequently than any of the other 15 genes assessed. *TP53* was also observed to have the highest LOH rate (91–97%) compared with the other genes in all six tumour types, suggesting that TP53 mutations are likely to be an early event in these tumours.

Tumour samples with HRR mutations showed higher HRD-LOH and TMB scores compared with HRR wild-type samples (Supplementary Table 4, Supplementary Figs. 5 and 6) supporting a functional impact for LoF mutations in these candidate HRR genes. Given that TMB is associated to a response to immunotherapy, HRR mutations, could, therefore, potentially represent patient selection biomarkers for PARP inhibitors and immunotherapy, both alone and in combination. Approximately half of MSI-high samples carried a deleterious HRR mutation, while only a small proportion HRR mutant samples were MSI-high (Supplementary Table 6). Within MSI-high, samples with HRR mutation tend to have even higher TMB (Supplementary Fig. 9), suggesting that in MSI-high background, HRR mutation might further contribute to TMB.

Other studies have also assessed the profile of HRR mutations and measurements of DNA repair deficiency across tumour types [21, 39, 40]. Heeke et al showed that HRR mutations were seen in 17.4% of tumours across 21 cancer lineages, most commonly in endometrial, biliary tract, bladder, hepatocellular, gastroesophageal and ovarian cancer [39]. Kraya et al [40] reported that in *BRCA1/2* breast cancers that HRD scores and hormone receptor subtype were predictive of immunogenicity resulting from their increased genomic instability making them theoretically more sensitive to checkpoint inhibitors, although in practice only 20% of patients with *BRCA1*/2 mutations respond to PD-1/PD-L1 inhibition suggesting that a combination of factors involving *BRCA1*/2 status, HRD and hormone receptor status may more effectively predict breast cancer patients who will respond to checkpoint inhibitors than any one factor alone. Additionally, Johnson *et al* [41] recently reported that selective pressure for bi-allelic inactivation and therefore sensitivity to PARP inhibition was observed only in BRCA-associated tumour types of breast, ovary, prostate or pancreatic cancers, and BRCA mutations mainly appeared biologically neutral in patients with non-BRCA-associated cancer. However, as to be expected in tumours where BRCA mutations are rare and PARP inhibitors are not approved as treatment, the very small patient numbers assessed (14 BRCAm vs. 20 BRCAwt), combined with uncertainty over patient sensitivity to previous therapies and type of PARP inhibitor administered, bring a degree of uncertainty to these findings.

We also examined the prevalence of BRCA and HRR mutations in HER2-amplified breast cancer, and found 5% of those carried mutations in BRCA, and another 16% carried mutation in non-BRCA HRR genes. Given that olaparib has been approved for metastatic HER2- breast cancer with a germline BRCA mutation [42], it is reasonable to speculate that adding PARPi to treatments of HER2+ breast cancer might be beneficial in the presence of somatic BRCA or HRR mutations.

Reversion mutations are a known resistance mechanism to both platinum-based chemotherapy and PARP inhibitors. Even though we did not have treatment information for the samples in this cohort, it is known that many samples were not treatment-naïve. We examined all mutations reported out of 4583 total patients with deleterious BRCA mutations, excluding gene deletions and rearrangements, and identified 191 potential reversion mutations in 157 samples (Fig. 4 and Supplementary Table 5) from all six different tumour types; bladder, lung, ovarian, breast, pancreas, and prostate, four of which have positive Phase III trials for PARP inhibitors. We also observed that in the breast and ovarian tumour samples, where a significant number of samples with reversion BRCA mutation were detected, the samples have significantly higher gLOH scores than those without a reversion mutation. This supports the expectation that reversion BRCA mutations will not reverse the genomic scars that are already present (Supplementary Fig. 9). The number of these reversion mutations in tissue samples suggests they deserve more attention in clinical reporting, as they may be predicted as variants of uncertain significance by current clinical assays, thus not actionable. This is especially important as PARP inhibitors are becoming more widely used in the clinic and different results may be expected from diagnostic and post-treatment tumour samples. The detection of reversion mutation would suggest that patients will be unlikely to respond to those therapies and different treatments need to be considered, or at least that the patients need to be closely monitored. We believe that putative reversion mutations reported here are a lower bound, as it is still a challenge to call large indels in clinical assays, and some reversion mutations might not be easily interpreted. In addition, given that many these putative reversion mutations are fairly large, it is important to choose assays or variant callers, such as VarDict, that have the capability to call such large variants.

Limitations to our study include awareness that the genes involved in HRR are not yet comprehensively defined, and only a subset of those genes are included in this study. In addition, an established way to measure HRD has not been standardized; different studies have measured HRD using different assays including assessment of TAI, LST and loss of heterozygosity or a combination of these methods. Another limitation is that we only analysed *BRCA1* and *BRCA2* genes in TCGA cohorts due to resource limitations, and the lower incidence of the remaining HRR genes as the cohorts are small. The larger Foundation Medicine cohorts, on the other hand, were derived from relevant clinical testing using formalin-fixed paraffin-embedded tissue samples and should more accurately reflect the true prevalence for patients in the clinics.

A notable finding in our study is the identification of putative BRCA reversion mutations in tissue from bladder, lung, ovarian, breast, pancreatic, and prostate cancers. Even though we do not know whether these samples were treated by platinum or PARP inhibitor, the presence of putative reversion mutations suggest that those tumours still relied on BRCA function to survive, and reversion mutations provide a way for them to escape therapeutic pressure. It has been demonstrated that tumours with BRCA reversions are unlikely to respond to PARP inhibition and may be annotated as variants of uncertain significance; however, it highlights the importance of their correct annotation in clinical next-generation sequencing testing reports so that more appropriate therapies can be selected.

## Conclusions

The similarity between somatic and germline mutations for breast cancers compared with ovarian cancer shown in this study provides further evidence that personalized anti-cancer therapies known to be active against germline mutations may also show clinical activity against somatic BRCA tumours. The data presented here will facilitate future research into the efficacy of precision oncology treatments, including PARP and immune checkpoint inhibitors.

## Supplementary Information

**Additional file 1.**

## Data Availability

The data that support the findings of this study are available from TCGA and FMI but restrictions apply to the availability of these data, which were used under license for the current study, and so are not publicly available.

## Abbreviations

BRCAm: Mutations in *BRCA1* and *BRCA2*
CI: Confidence interval
DDR: DNA damage response
DSB: Double-strand break
ER+: Oestrogen-receptor positive
ER-: Oestrogen-receptor negative
HER2: Human epidermal growth factor receptor 2
HER2+: Human epidermal growth factor receptor 2 positive
HER2-: Human epidermal growth factor receptor 2 negative
HRD: Homologous recombination deficiency
HRR: Homologous recombination repair
HRRm: Homologous recombination repair mutation
HRRwt: Homologous recombination repair without mutation
IGV: Integrative Genomics Viewer
gLOH: Genomic loss of heterozygosity
LoF: Loss of function
LOH: Loss of heterozygosity
LST: Large-scale transition
MSI: Microsite instability
OR: Odds ratio
OS: Overall survival
PARP: Poly(ADP-ribose) polymerase
PARPi: Poly(ADP-ribose) polymerase inhibitor
PD-1: Programmed cell death 1
PD-L1: Programmed death ligand 1
PFS: Progression-free survival
SGZ: Somatic-germline zygosity
SNV: Single nucleotide variation
TAI: Telomeric allelic imbalance
TCGA: The Cancer Genome Atlas
TMB: Tumour mutation burden
TNBC: Triple-negative breast cancer
