# Supplementary material for: Landscape of homologous recombination deficiencies in solid tumours: analyses of two independent genomic datasets
Source: BMC Cancer. 2022 Jan 3;22:13. doi: 10.1186/s12885-021-09082-y (PMC8722117; doi:10.1186/s12885-021-09082-y)
Supplement: Supplementary file 1 — Additional file 1. [file 12885_2021_9082_MOESM1_ESM.pdf]

## Supplement to:

# Landscape of homologous recombination deficiencies in solid tumours: analyses of two independent genomic datasets

Zhongwu Lai, Matthew Brosnan, Ethan Sokol, Mingchao Xie, Jonathan R. Dry, Elizabeth A. Harrington, J. Carl Barrett, Darren Hodgson

## Contents

Supplementary Figure 1 ... 2
Supplementary Figure 2 ... 2
Supplementary Figure 3 ... 3
Supplementary Figure 4 ... 4
Supplementary Figure 5 ... 5
Supplementary Figure 6 ... 6
Supplementary Figure 7 ... 7
Supplementary Figure 8 ... 8
Supplementary Figure 9 ... 9
Supplementary Table 1 ... 10
Supplementary Table 2 ... 10
Supplementary Table 3 ... 10
Supplementary Table 4 ... 11
Supplementary Table 5 ... 11
Supplementary Table 6 ... 11
Supplementary Table 7 ... 12
Supplementary text ... 13

**Supplementary Figure 1**

**HRD scores by BRCA status in TCGA ovarian and breast cancer cohorts.**

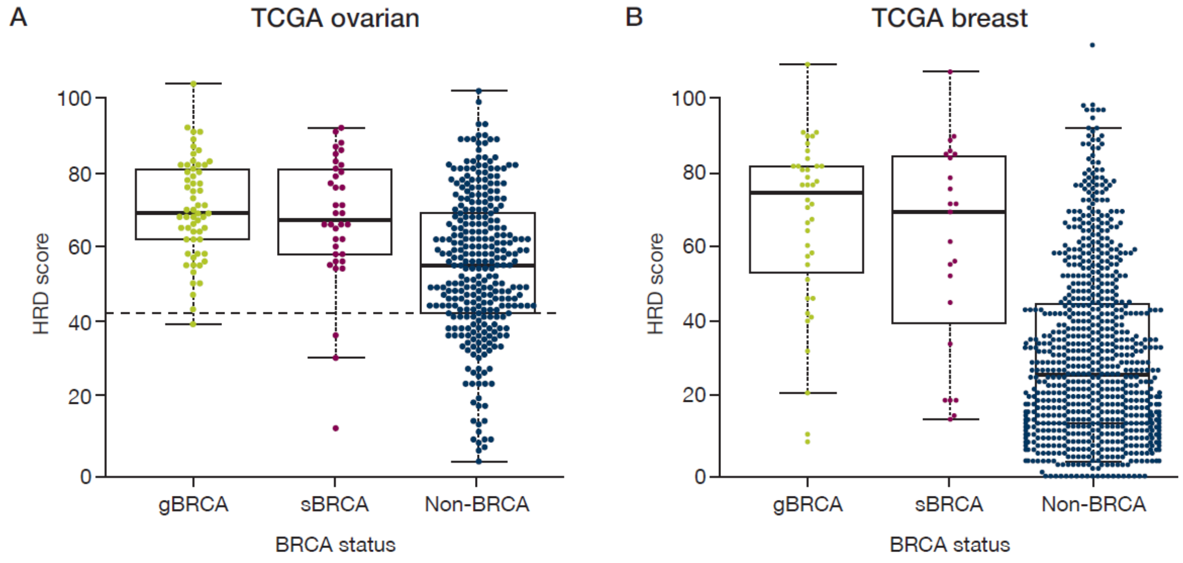


HRD, homologous recombination deficiency; TCGA, The Cancer Genome Atlas.

**Supplementary Figure 2**

**Age of diagnosis by BRCA status in TCGA ovarian and breast cancer cohorts.**

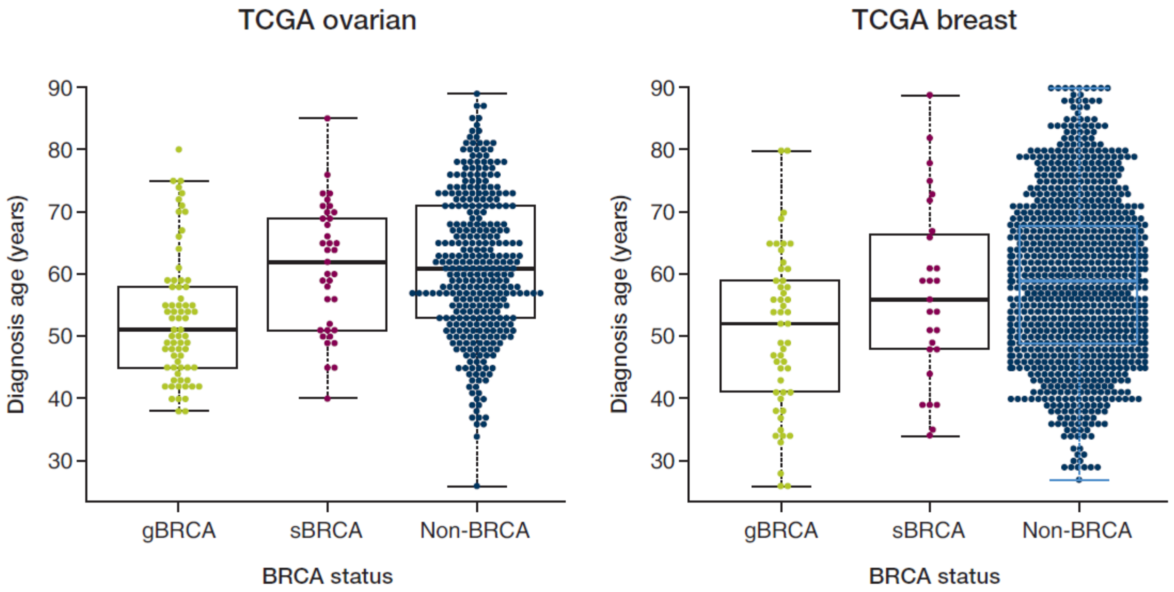


TCGA, The Cancer Genome Atlas.

Patients with germline BRCA mutations, who were born with the mutation, would develop tumours at younger ages than those with somatic BRCA mutations, which have to be acquired later in life. Indeed, we found patients with germline BRCA mutations were diagnosed at a younger age than patients with somatic BRCA mutations in both the ovarian cancer cohort (median 52 and 63, respectively, and Mann-Whitney U test *P* value of 0.003) and the breast cancer cohort (median 52 and 56 years, respectively, and Mann-Whitney U test *P* value of 0.078). The reason for this observation is suggested to be due to patients carrying a gBRCA mutation having a defect in DNA repair in every cell when

they were born, and thus are more likely to acquire secondary cancer-causing mutations at early stage of their lives. Patients with somatic BRCA mutations had similar diagnostic age as those without a BRCAm (61 and 59, respectively), with t-test *P* values of 0.98 and 0.50 for ovarian and breast cancers, respectively.

**Supplementary Figure 3**

**Genome wide-LOH scores by *BRCA1* and *BRCA2* status in Foundation Medicine ovarian and breast cancer mutation cohorts.**

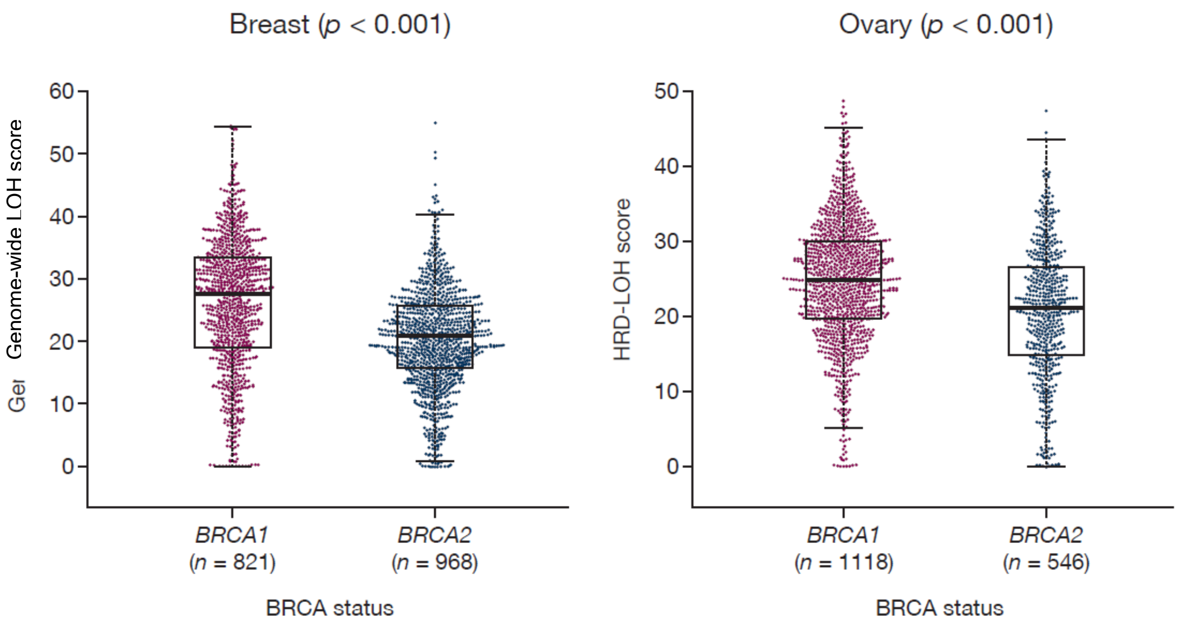


LOH, loss of heterozygosity.

## Supplementary Figure 4

**Oncoprint analysis of Foundation Medicine ovarian and breast cancer cohorts.**

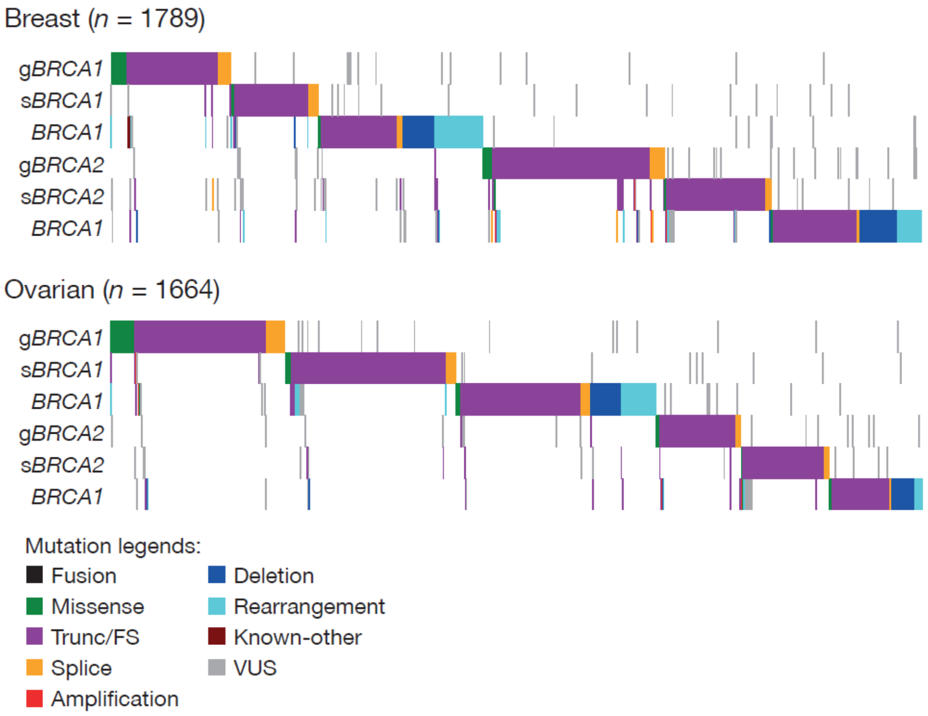


g*BRCA1*, g*BRCA2*: samples with germline *BRCA1* or *BRCA2* mutations; s*BRCA1*, s*BRCA2*: samples with somatic *BRCA1* or *BRCA2* mutations; *BRCA1*, *BRCA2*: samples whose mutation origin cannot be determined by SGZ algorithm, including all deletions and rearrangements. VUS, variant of unknown significance.

The figure shows that the vast majority of *BRCA1* and *BRCA2* mutations are truncating mutations, and that not only are *BRCA1* and *BRCA2* mutations mutually exclusive, but germline and somatic mutations are also mutually exclusive to either other.

**Supplementary Figure 5**

**HRD-LOH scores in patients with and without HRR gene mutations, including BRCA, in the Foundation Medicine dataset.**

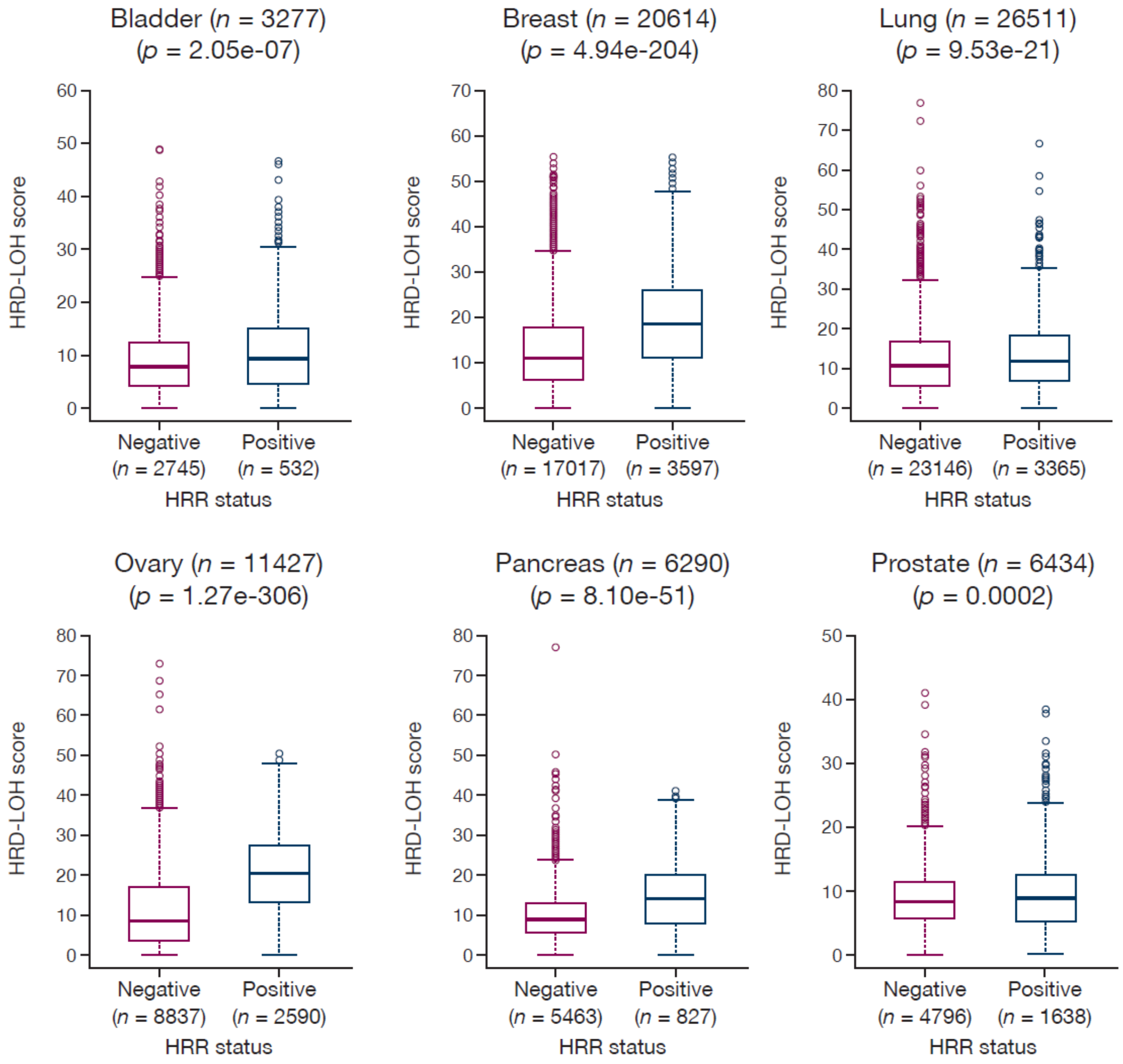


HRD, homologous recombination deficiency; HRR, homologous recombination repair; LOH, loss of heterozygosity.

## Supplementary Figure 6

**TMB in patients with and without HRR gene mutations in the Foundation Medicine dataset.**

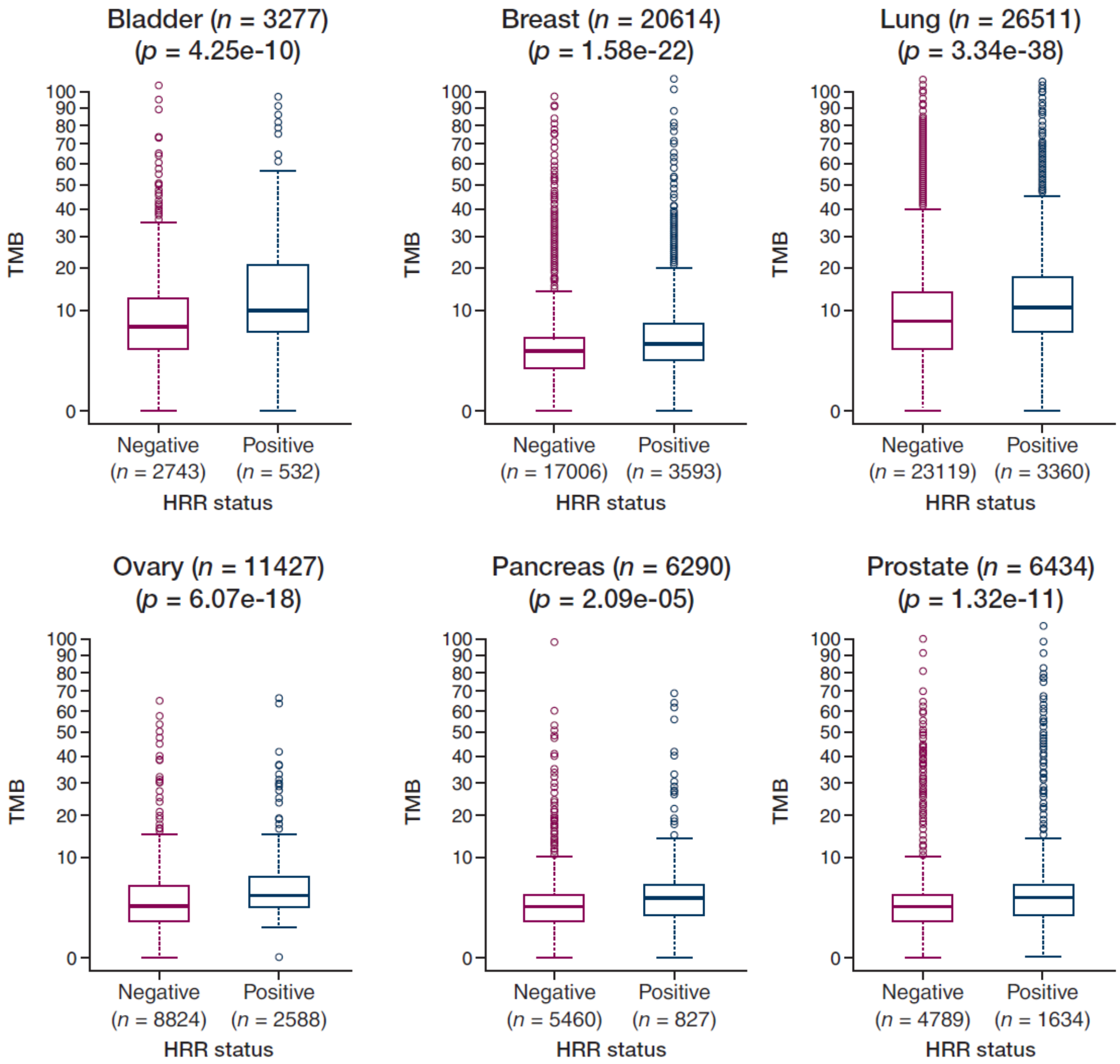


HRR, homologous recombination repair; TMB, tumour mutation burden.

**Supplementary Figure 7**

**HRD-LOH scores by HRR gene mutation – heterozygous (mono-allelic loss without LOH) versus homozygous (bi-allelic loss of function with LOH) versus compound heterozygous (bi-allelic loss of function without LOH) versus unknown versus wild-type – in Foundation Medicine dataset (a) bladder, (b) breast, (c) lung, (d) ovarian, (e) pancreas and (f) prostate tumour samples.**

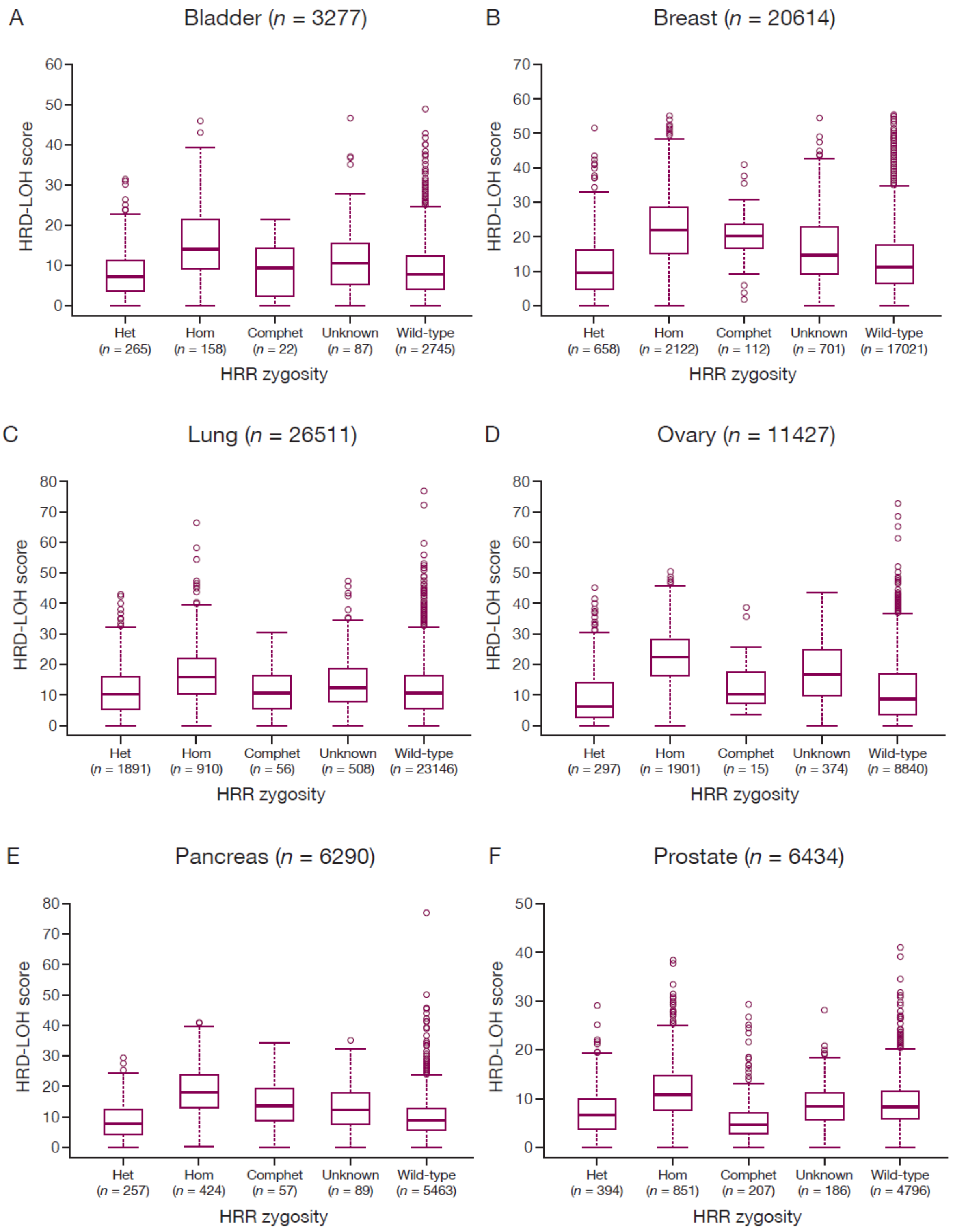


HRD, homologous recombination deficiency; HRR, homologous recombination repair; LOH, loss of heterozygosity.

**Supplementary Figure 8**

**In breast and ovarian samples with BRCA mutations, gLOH scores were compared between those with BRCA reversion mutations and those without. Samples with reversion mutations detected have significantly higher gLOH scores with *p* values of 6.02e-5 and 7.89e-5, respectively for breast and ovarian.**

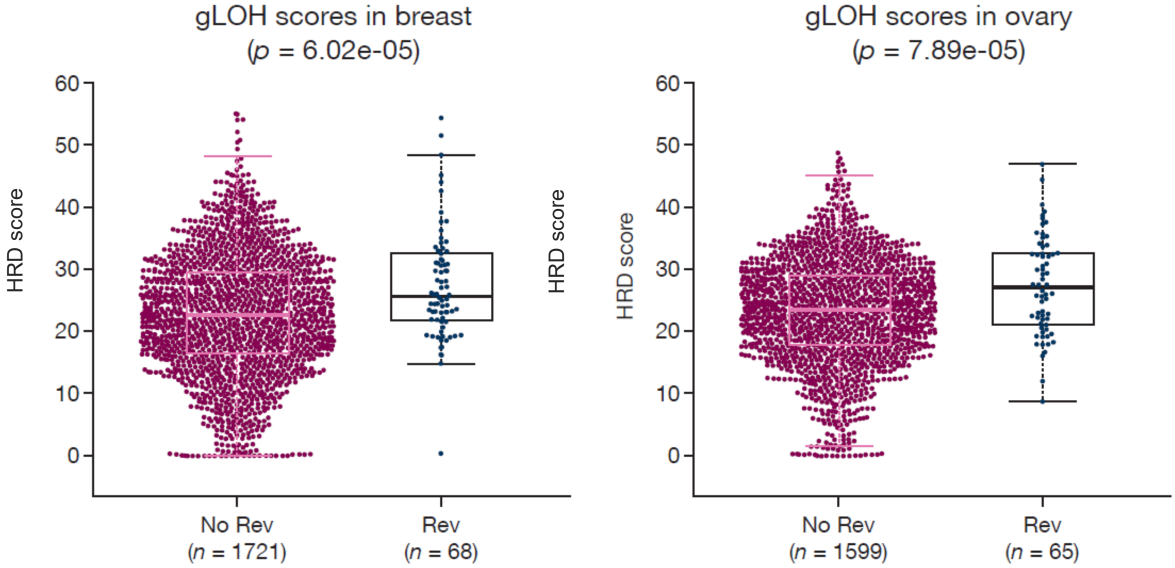


gLOH, genomic loss of heterozygosity; HRD, homologous recombination deficiency; Rev, reversion mutation.

**Supplementary Figure 9**

**In MSI-high subset of samples, those with HRR mutation detected have consistent trend of higher TMB than those without, though most do not achieve statistical significance.**

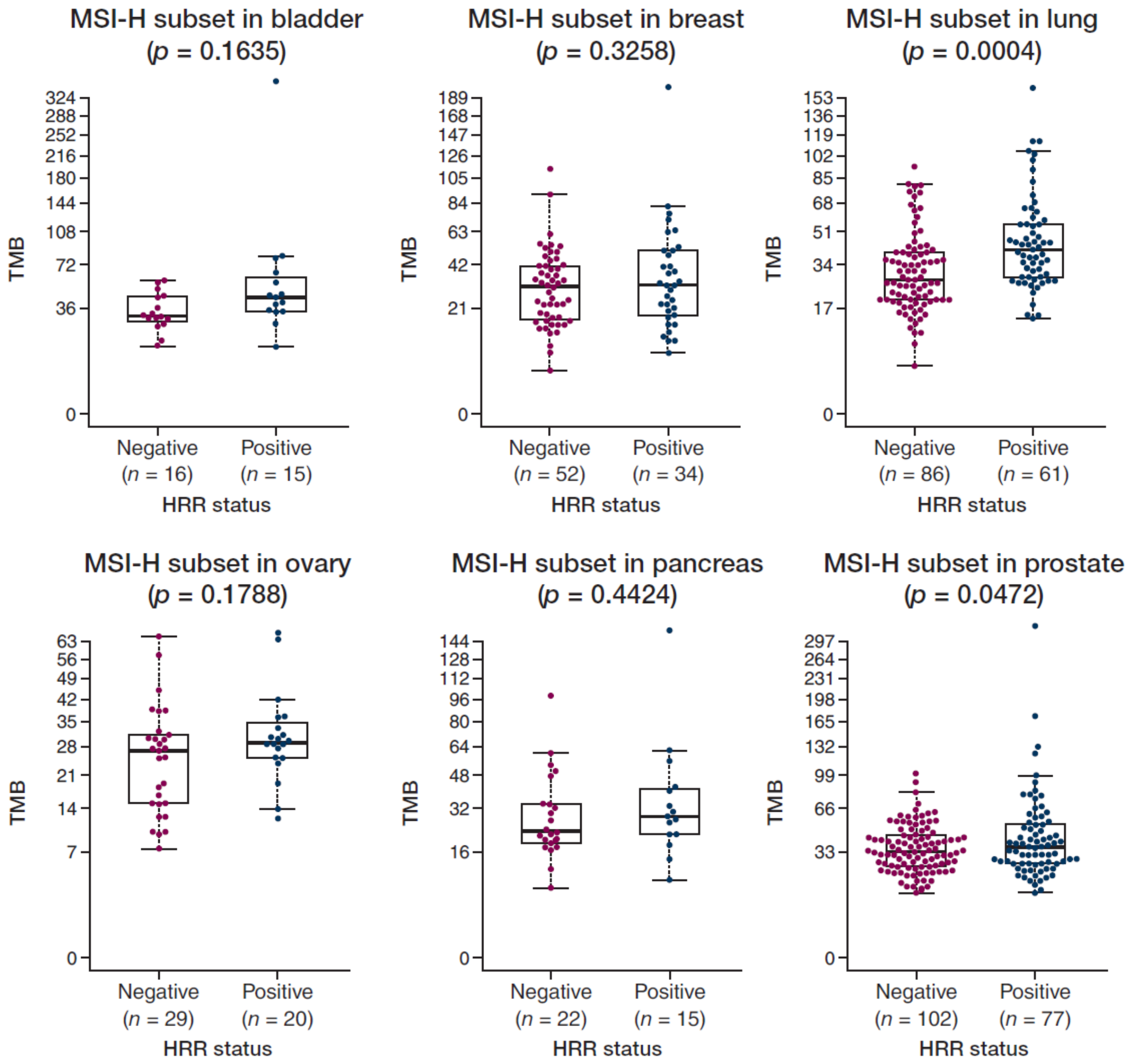


HRR, homologous recombination repair; MSI-H, microsatellite instability-high; TMB, tumour mutation burden.

**Supplementary Table 1**

**Bi-allelic loss of BRCA in ER+ and ER– breast cancer in the TCGA cohort.**

| | **ER+** | **ER-** | **ER unknown** |
|---|---|---|---|
| Homozygous (bi-allelic loss with LOH), *n* (%) | 33 (85) | 27 (90) | 7 (100) |
| Composite heterozygous (without LOH but considered bi-allelic loss), *n* (%) | 0 | 2 (7) | 0 |
| Heterozygous (mono-allelic without LOH), *n* (%) | 4 (15) | 1 (3) | 0 |

ER, oestrogen receptor; LOH, loss of heterozygosity; TCGA, The Cancer Genome Atlas.

**Supplementary Table 2**

**HRR gene mutation frequency in the Foundation Medicine dataset across six tumour types.**

| | **Bladder (*n* = 3277)** | **Breast (*n* = 20,614)** | **Lung (*n* = 26,511)** | **Ovarian (*n* = 11,427)** | **Pancreatic (*n* = 6290)** | **Prostate (*n* = 6434)** |
|---|---|---|---|---|---|---|
| HRR*, % | 16.2 | 17.4 | 12.7 | 22.7 | 13.1 | 25.5 |
| TP53, % | 62.6 | 54.8 | 72.2 | 79.0 | 77.0 | 44.3 |
| MSI-H, % | 0.9 | 0.4 | 0.6 | 0.4 | 0.6 | 2.8 |

*HRR is defined as deleterious mutations in any of 15 genes in the panel: BRCA1, BRCA2, ATM, CDK12, RAD15B, RAD51C, RAD54L, RAD51D, BRIP1, FANCI, FANCL, PALB2, BARD1, CHEK1, CHEK2. HRR, homologous recombination repair; MSI-H, microsatellite instability-high.

**Supplementary Table 3**

**Bi-allelic loss of function rates of HRR gene mutations in the Foundation Medicine dataset.**

| | **Bladder (*n* = 3277)** | **Breast (*n* = 20,614)** | **Lung (*n* = 26,511)** | **Ovarian (*n* = 11,427)** | **Pancreatic (*n* = 6290)** | **Prostate (*n* = 6434)** |
|---|---|---|---|---|---|---|
| HRR* bi-allelic rate, % | 40.4 | 77.2 | 33.8 | 86.6% | 65.2 | 72.9 |

*A HRR mutation was defined as a deleterious mutation in any of the 15 genes in the panel. HRR, homologous recombination repair.

**Supplementary Table 4**

**Mean TMB and percent genome wide-LOH scores in the Foundation Medicine dataset.**

| Location | TMB (HRRm) | TMB (HRRwt) | *P* value (Mann-Whitney U test) | LOH score (HRRm) | LOH score (HRRwt) | *P* value (t-test) |
|---|---|---|---|---|---|---|
| Bladder | 14.9 | 9.6 | <0.0001 | 11.2 | 9.1 | <0.0001 |
| Breast | 6.2 | 4.8 | <0.0001 | 19.1 | 13.1 | <0.0001 |
| Lung | 14.4 | 10.5 | <0.0001 | 13.1 | 11.6 | <0.0001 |
| Ovarian | 4.8 | 3.4 | <0.0001 | 20.3 | 11.3 | <0.0001 |
| Pancreatic | 4.8 | 2.9 | <0.0001 | 14.6 | 9.7 | <0.0001 |
| Prostate | 4.0 | 2.8 | <0.0001 | 9.6 | 9.0 | 0.0002 |

*P* values were not corrected for multiple testing; MSI-H samples have been removed for TMB assessment, but kept in LOH assessment. HRRm, mutated homologous recombination repair; HRRwt, wild-type homologous recombination repair; LOH, loss of heterozygosity; MSI-H, microsatellite instability-high; TMB, tumour mutation burden.

**Supplementary Table 5**

**The 47 samples with likely reversion mutations detected are listed, including the original sensitizing functional mutations.**

(Shown on page 14)

**Supplementary Table 6**

**MSI-H and HRR mutations in the Foundation Medicine dataset**

| Disease | MSI-H, *n* | HRRm, *n* | MSI-H + HRRm, *n* | %HRRm in MSI-H | %MSI-H in HRRm |
|---|---|---|---|---|---|
| Bladder | 31 | 532 | 15 | 48 | 2.8 |
| Breast | 86 | 3597 | 34 | 40 | 0.9 |
| Lung | 147 | 3365 | 61 | 41 | 1.8 |
| Ovarian | 49 | 2590 | 20 | 41 | 0.8 |
| Pancreatic | 37 | 827 | 15 | 41 | 1.8 |
| Prostate | 179 | 1638 | 77 | 43 | 4.7 |

HRRm, mutated homologous recombination repair; MSI-H, microsatellite instability-high; TMB, tumour mutation burden.

Analysis of the relationship between MSI-high (MSI-H) and HRR showed that about half of MSI-H samples carry deleterious HRR mutations; however, only a fraction of samples with deleterious HRR mutations are MSI-H (1–4%). Interestingly, within these MSI-H tumours, those with a HRRm detected consistently tend to have even higher TMB than those without (Supplementary Fig. 9), suggesting HRR mutations might further contribute to the TMB even in MSI-H background.

**Supplementary Table 7**

**Breakdown of somatic, germline, or unknown origins for deleterious mutations in HRR genes across tumour types. Numbers are patient counts. A patient is classified as germline if at least one deleterious mutation for the gene is classified as germline by SGZ algorithm, as somatic if all deleterious mutations of the gene are classified as somatic by SGZ algorithm. Otherwise, the patient will be classified as unknown**

| HRR gene | Status | Bladder (*n*=3277) | Breast (*n*=20614) | Lung (*n*=26511) | Ovary (*n*=11427) | Pancreas (*n*=6290) | Prostate (*n*=6434) |
|---|---|---|---|---|---|---|---|
| ***ATM*** | Germline | 12 | 135 | 177 | 42 | 76 | 62 |
| | Somatic | 81 | 149 | 598 | 95 | 100 | 151 |
| | Unknown | 32 | 140 | 234 | 72 | 42 | 137 |
| ***BARD1*** | Germline | 2 | 25 | 26 | 10 | 10 | 4 |
| | Somatic | 4 | 32 | 87 | 10 | 3 | 7 |
| | Unknown | 9 | 47 | 37 | 12 | 6 | 14 |
| ***BRCA1*** | Germline | 8 | 266 | 55 | 358 | 47 | 11 |
| | Somatic | 22 | 191 | 135 | 350 | 15 | 21 |
| | Unknown | 36 | 364 | 180 | 410 | 43 | 47 |
| ***BRCA2*** | Germline | 13 | 404 | 125 | 177 | 139 | 152 |
| | Somatic | 45 | 242 | 240 | 184 | 64 | 124 |
| | Unknown | 39 | 343 | 169 | 199 | 79 | 342 |
| ***BRIP1*** | Germline | 8 | 35 | 52 | 52 | 17 | 9 |
| | Somatic | 10 | 31 | 131 | 17 | 9 | 14 |
| | Unknown | 14 | 120 | 51 | 46 | 8 | 11 |
| ***CDK12*** | Germline | 3 | 19 | 20 | 17 | 6 | 28 |
| | Somatic | 22 | 40 | 97 | 110 | 9 | 246 |
| | Unknown | 32 | 217 | 93 | 123 | 13 | 132 |
| ***CHEK1*** | Germline | 2 | 10 | 13 | 4 | 1 | 0 |
| | Somatic | 6 | 6 | 48 | 2 | 1 | 3 |
| | Unknown | 4 | 6 | 11 | 2 | 0 | 0 |
| ***CHEK2*** | Germline | 18 | 134 | 91 | 26 | 26 | 32 |
| | Somatic | 17 | 67 | 115 | 17 | 4 | 14 |
| | Unknown | 19 | 112 | 91 | 47 | 17 | 33 |
| ***FANCI*** | Germline | 0 | 8 | 12 | 7 | 0 | 0 |
| | Somatic | 3 | 16 | 36 | 8 | 2 | 5 |
| | Unknown | 2 | 11 | 17 | 9 | 2 | 2 |
| ***FANCL*** | Germline | 10 | 45 | 60 | 18 | 10 | 18 |
| | Somatic | 11 | 35 | 66 | 19 | 11 | 18 |
| | Unknown | 18 | 55 | 97 | 53 | 16 | 23 |
| ***PALB2*** | Germline | 5 | 129 | 37 | 23 | 26 | 24 |
| | Somatic | 11 | 69 | 98 | 9 | 11 | 15 |
| | Unknown | 14 | 83 | 54 | 32 | 13 | 15 |
| ***RAD51B*** | Germline | 1 | 15 | 10 | 7 | 5 | 2 |
| | Somatic | 1 | 14 | 26 | 5 | 1 | 1 |
| | Unknown | 23 | 89 | 40 | 18 | 8 | 25 |
| ***RAD51C*** | Germline | 0 | 16 | 11 | 25 | 9 | 0 |
| | Somatic | 6 | 24 | 19 | 9 | 4 | 2 |
| | Unknown | 3 | 38 | 22 | 32 | 12 | 5 |

| | | | | | | | |
|---|---|---|---|---|---|---|---|
| ***RAD51D*** | Germline | 0 | 13 | 13 | 26 | 5 | 2 |
| | Somatic | 2 | 11 | 33 | 10 | 1 | 1 |
| | Unknown | 2 | 16 | 20 | 29 | 3 | 4 |
| ***RAD54L*** | Germline | 1 | 9 | 26 | 7 | 5 | 4 |
| | Somatic | 8 | 14 | 28 | 6 | 1 | 3 |
| | Unknown | 2 | 16 | 13 | 10 | 3 | 7 |

HRR, homologous recombination repair

## Supplementary text

### Putative reversion mutations

Of the putative reversion mutations, 173 are either large deletions that delete the original sensitizing truncation or frameshift mutations, or SNVs that convert nonsense into missense (e.g., BRCA1 c.5444G>A W1815* to c.5443_5444TG>AA W1815K) or known missense to unknown missense (e.g., BRCA1 c.5324T>G M1775R to c.5324_5325TG>GT M1775S). The remaining 18 reversion mutations are frameshift causing mutations adjacent to the original sensitizing mutation, but when combined, will restore the reading frame. It is worth noting that for those SNV reversion mutations, the reversion mutations often happen next to the original mutation, thus becoming two or three nucleotide variants. There are two known deleterious mutations, *BRCA1* M1775R (c.5324T>G) and A1708E (c.5123C>A), we identified the candidate reversion mutations as *BRCA1* M1775S (c.5324_5325TG>GT) and M1708G (c.5123_5124CG>GT), respectively. The reversion mutations change to a different amino acid. This suggests that they are not hotspot, and only certain amino acid can cause loss of function. Furthermore, in the vast majority of cases, the reversion mutations have lower allele frequencies (AF) than the original sensitizing mutations.

| Tissue | Sample | Gene | variant_type | fmi_reportable_status | hrr | variant_cds | variant_pe | variant_af | chrom | pos | somatic_germline_status | zygosity | Reversion |
|---|---|---|---|---|---|---|---|---|---|---|---|---|---|
| bladder | XRN:0V4MHY | BRCA1 | SV | likely | TRUE | 3477_3479AAA>C | K1160fs*4 | 0.652 | chr17 | 41244069 | unknown | homozygous | Sen |
| bladder | XRN:0V4MHY | BRCA1 | SV | likely | FALSE | 3456_3503del48 | L1153_N1168del | 0.1773 | chr17 | | unknown | | Rev |
| breast | XRN:16G7UN | BRCA1 | SV | likely | TRUE | 3598C>T | Q1200* | 0.7815 | chr17 | 41243950 | germline | homozygous | Sen |
| breast | XRN:16G7UN | BRCA1 | SV | likely | TRUE | 2591_4096+17del1523 | splice site 2591_4096+17del1523 | 0.0512 | chr17 | | unknown | | Rev |
| breast | XRN:YAL8EH | BRCA1 | SV | likely | TRUE | 2504_2505insAAGTATCCATTGGGACA | H835fs*17 | 0.291 | chr17 | 41245043 | unknown | homozygous | Sen |
| breast | XRN:YAL8EH | BRCA1 | SV | likely | FALSE | 2099_2713del615 | L700_E904del | 0.4113 | chr17 | | unknown | | Rev |
| breast | XRN:PUDBAP | BRCA1 | SV | unknown | FALSE | 3228_3234AGGGCCA>GGCC | G1077_P1078>A | 0.3986 | chr17 | 41244314 | unknown | homozygous | Rev |
| breast | XRN:PUDBAP | BRCA1 | SV | likely | TRUE | 3228_3229delAG | G1077fs*8 | 0.1729 | chr17 | 41244318 | germline | not in tumor | Sen |
| breast | XRN:S7UTVL | BRCA1 | SV | unknown | FALSE | 2786_3100del315 | F929_N1034>Y | 0.1494 | chr17 | | unknown | | Rev |
| breast | XRN:S7UTVL | BRCA1 | SV | likely | TRUE | 2834_2835delGT | S945fs*6 | 0.8407 | chr17 | 41244712 | unknown | het | Sen |
| breast | XRN:X12PKP | BRCA1 | SV | likely | TRUE | 1736_2135del400 | A579fs*24 | 0.1899 | chr17 | | unknown | | Rev |
| breast | XRN:X12PKP | BRCA1 | SV | likely | TRUE | 1713_1717delAGAAT | E572fs*12 | 0.7903 | chr17 | 41245830 | germline | homozygous | Sen |
| breast | XRN:X0Q7WC | BRCA1 | SV | likely | FALSE | 2674_3528del855 | L892_V1176del | 0.18 | chr17 | | unknown | | Rev |
| breast | XRN:X0Q7WC | BRCA1 | SV | likely | FALSE | 2921_2938delTACAAAACCCATATCGTA | Q975_I980del | 0.09 | chr17 | 41244609 | somatic | homozygous | Rev |
| breast | XRN:X0Q7WC | BRCA1 | SV | likely | TRUE | 2934T>G | Y978* | 0.5 | chr17 | 41244614 | unknown | homozygous | Sen |
| breast | XRN:VMUS6A | BRCA1 | SV | likely | FALSE | 1086_1211del126 | N363_E404del | 0.2817 | chr17 | | unknown | . | Rev |
| breast | XRN:VMUS6A | BRCA1 | SV | likely | TRUE | 1121_1121delC | T374fs*2 | 0.4518 | chr17 | 41246426 | unknown | homozygous | Sen |
| breast | XRN:WLTCTH | BRCA1 | SV | likely | FALSE | 5266_5271delCAGGAC | Q1756_D1757del | 0.1919 | chr17 | 41209074 | unknown | homozygous | Rev |
| breast | XRN:WLTCTH | BRCA1 | SV | likely | TRUE | 5266_5267insC | Q1756fs*74 | 0.7014 | chr17 | 41209079 | unknown | homozygous | Sen |
| breast | XRN:P3PANB | BRCA1 | SV | likely | TRUE | 1789G>T | E597* | 0.6174 | chr17 | 41245759 | unknown | homozygous | Sen |
| breast | XRN:P3PANB | BRCA1 | SV | likely | FALSE | 1750_1791del42 | A584_E597del | 0.5164 | chr17 | | unknown | . | Rev |
| breast | XRN:4CYL9C | BRCA1 | SV | likely | FALSE | 2057_2216>GACAAGTAAAAGACATGAC | E686_K739>GQVKDMT | 0.1714 | chr17 | 41245329 | germline | not in tumor | Rev |
| breast | XRN:4CYL9C | BRCA1 | SV | likely | TRUE | 2057_2060delAACA | E686fs*14 | 0.4154 | chr17 | 41245487 | unknown | homozygous | Sen |
| breast | XRN:SLWBJM | BRCA1 | SV | unknown | FALSE | 5267_5274AGGACAGA>CAGGACAG | Q1756_R1758>PGQ | 0.1367 | chr17 | 41209072 | germline | not in tumor | Rev |
| breast | XRN:SLWBJM | BRCA1 | SV | likely | TRUE | 5266_5267insC | Q1756fs*74 | 0.4468 | chr17 | 41209079 | unknown | homozygous | Sen |
| breast | XRN:66BFNJ | BRCA1 | SV | likely | FALSE | 2293_2295GAG>TAC | E765Y | 0.2263 | chr17 | 41245253 | unknown | homozygous | Rev |
| breast | XRN:66BFNJ | BRCA1 | SV | likely | TRUE | 2293G>T | E765* | 0.6057 | chr17 | 41245255 | somatic | homozygous | Sen |
| breast | XRN:2LXTB3 | BRCA1 | SV | likely | FALSE | 3678_3827del150 | F1226_I1275del | 0.257 | chr17 | | unknown | . | Rev |
| breast | XRN:2LXTB3 | BRCA1 | SV | likely | TRUE | 3748G>T | E1250* | 0.6501 | chr17 | 41243800 | unknown | homozygous | Sen |
| breast | XRN:SANFC7 | BRCA1 | SV | known | TRUE | 5324T>G | M1775R | 0.49 | chr17 | 41203088 | unknown | homozygous | Sen |
| breast | XRN:SANFC7 | BRCA1 | SV | likely | FALSE | 5324_5325TG>GT | M1775S | 0.39 | chr17 | 41203087 | unknown | homozygous | Rev |
| breast | XRN:61L4VG | BRCA1 | SV | likely | TRUE | 3700_3704delGTAAA | V1234fs*8 | 0.2113 | chr17 | 41243843 | unknown | homozygous | Sen |
| breast | XRN:61L4VG | BRCA1 | SV | unknown | FALSE | 3700_3721>CAATACTATG | V1234_S1241>QYYA | 0.5987 | chr17 | 41243827 | unknown | homozygous | Rev |
| breast | XRN:61L4VG | BRCA1 | SV | likely | TRUE | 3624_3639delATTAGAGTCCTCAGAA | L1209fs*21 | 0.1331 | chr17 | 41243908 | germline | not in tumor | Rev |
| breast | XRN:XTF8U4 | BRCA1 | SV | known | TRUE | 280C>T | Q94* | 0.35 | chr17 | 41256906 | unknown | homozygous | Sen |
| breast | XRN:XTF8U4 | BRCA1 | SV | likely | FALSE | 280_282CAG>TAC | Q94Y | 0.2 | chr17 | 41256904 | germline | not in tumor | Rev |
| breast | XRN:XTF8U4 | BRCA1 | SV | likely | FALSE | 280_281CA>TC | Q94S | 0.1 | chr17 | 41256905 | unknown | homozygous | Rev |
| breast | XRN:MKKK7S | BRCA1 | SV | likely | TRUE | 4116_4117delTG | C1372fs*1 | 0.3141 | chr17 | 41243028 | germline | not in tumor | Sen |
| breast | XRN:MKKK7S | BRCA1 | SV | likely | FALSE | 4101_4118delAGCAGCATCTGGGTGTGA | A1368_E1373del | 0.2836 | chr17 | 41243027 | germline | not in tumor | Rev |
| breast | XRN:231W8F | BRCA1 | SV | likely | TRUE | 2456_2456delC | D821fs*25 | 0.69 | chr17 | 41245091 | unknown | homozygous | Sen |
| breast | XRN:231W8F | BRCA1 | SV | unknown | FALSE | 2397_2510del114 | N799_V837>N | 0.05 | chr17 | | unknown | . | Rev |
| breast | XRN:QAG2VJ | BRCA1 | SV | likely | TRUE | 2806_2809delGATA | D936fs*63 | 0.686 | chr17 | 41244738 | germline | homozygous | Sen |
| breast | XRN:QAG2VJ | BRCA1 | SV | likely | FALSE | 2806_2840>AGCCAGTTGATAATGCCAAATGT | D936_K947>SQLIMPNV | 0.4847 | chr17 | 41244708 | somatic | homozygous | Rev |
| breast | XRN:3D07LV | BRCA1 | SV | known | TRUE | 5123C>A | A1708E | 0.7444 | chr17 | 41215920 | somatic | homozygous | Sen |
| breast | XRN:3D07LV | BRCA1 | SV | likely | FALSE | 5123_5124CG>GT | A1708G | 0.0799 | chr17 | 41215919 | unknown | homozygous | Rev |
| breast | XRN:PTKK1Q | BRCA1 | SV | unknown | FALSE | 5161_5162CA>TT | Q1721L | 0.68 | chr17 | 41215381 | somatic | homozygous | Rev |
| breast | XRN:PTKK1Q | BRCA1 | SV | likely | TRUE | 5161C>T | Q1721* | 0.16 | chr17 | 41215382 | germline | not in tumor | Sen |
| breast | XRN:BA7GC0 | BRCA1 | SV | unknown | FALSE | 5505A>G | R1835R | 0.2791 | chr17 | 41197782 | unknown | homozygous | Rev |
| breast | XRN:BA7GC0 | BRCA1 | SV | known | TRUE | 5503C>T | R1835* | 0.5325 | chr17 | 41197784 | somatic | homozygous | Sen |
| breast | XRN:BA7GC0 | BRCA1 | SV | unknown | FALSE | 5503_5505CGA>TGG | R1835W | 0.2764 | chr17 | 41197782 | unknown | homozygous | Rev |
| breast | XRN:6KMA34 | BRCA1 | SV | likely | FALSE | 1954_2033>AAAAAAAGTACAACCAAATGCCA GTCAGGCAC | K652_R664>KKSTTKCQSGT | 0.1443 | chr17 | 41245514 | germline | not in tumor | Rev |
| breast | XRN:6KMA34 | BRCA1 | SV | known | TRUE | 1961delA | K654fs*47 | 0.5616 | chr17 | 41245586 | unknown | homozygous | Sen |
| lung | XRN:HEV47D | BRCA1 | SV | likely | TRUE | 3514_3515GA>TG | E1172* | 0.46 | chr17 | 41244034 | somatic | het | Sen |
| lung | XRN:HEV47D | BRCA1 | SV | unknown | FALSE | 3515A>G | E1172G | 0.45 | chr17 | 41244033 | somatic | het | Rev |
| ovary | XRN:SAKNPU | BRCA1 | SV | likely | TRUE | 2995_3001delCTAGAGG | L999fs*23 | 0.1957 | chr17 | 41244546 | germline | not in tumor | Sen |
| ovary | XRN:SAKNPU | BRCA1 | SV | likely | FALSE | 2995_3008CTAGAGGAAAACTT>AAAAC | L999_F1003>KT | 0.2233 | chr17 | 41244540 | germline | not in tumor | Rev |
| ovary | XRN:XRTCMH | BRCA1 | SV | likely | FALSE | 3462_3522>TTTA | L1154_S1174>FL | 0.2561 | chr17 | 41244029 | unknown | homozygous | Rev |
| ovary | XRN:XRTCMH | BRCA1 | SV | likely | TRUE | 3485delA | D1162fs*48 | 0.6316 | chr17 | 41244062 | somatic | homozygous | Sen |
| ovary | XRN:8420U7 | BRCA1 | SV | likely | TRUE | 5444G>A | W1815* | 0.2163 | chr17 | 41199683 | somatic | homozygous | Sen |
| ovary | XRN:8420U7 | BRCA1 | SV | unknown | FALSE | 5443_5444TG>AA | W1815K | 0.6 | chr17 | 41199683 | somatic | homozygous | Rev |
| ovary | XRN:SMWUX1 | BRCA1 | SV | likely | TRUE | 685delT | S229fs*5 | 0.5667 | chr17 | 41246862 | somatic | homozygous | Sen |
| ovary | XRN:SMWUX1 | BRCA1 | SV | likely | FALSE | 681_695delATTTTCTGAGACGGA | E227_T231del | 0.215 | chr17 | 41246852 | germline | not in tumor | Rev |
| ovary | XRN:61HM5Y | BRCA1 | SV | likely | TRUE | 2475delC | D825fs*21 | 0.5982 | chr17 | 41245072 | unknown | homozygous | Sen |
| ovary | XRN:61HM5Y | BRCA1 | SV | likely | TRUE | 2529_2554del26 | S844fs*1 | 0.143 | chr17 | | unknown | . | Rev |
| ovary | XRN:61HM5Y | BRCA1 | SV | likely | FALSE | 2448_2516del69 | G817_H839del | 0.0703 | chr17 | | unknown | . | Rev |
| ovary | XRN:61HM5Y | BRCA1 | SV | likely | FALSE | 2394_3272del879 | N799_P1091del | 0.0954 | chr17 | | unknown | . | Rev |
| ovary | XRN:VYQJ23 | BRCA1 | SV | likely | TRUE | 2681_2682delAA | K894fs*8 | 0.4723 | chr17 | 41244865 | somatic | homozygous | Sen |

| | | | | | | | | | | | | | |
|---|---|---|---|---|---|---|---|---|---|---|---|---|---|
| ovary | XRN:VYQJ23 | BRCA1 | SV | likely | FALSE | 2664_2729del66 | H888_N909del | 0.485 | chr17 | | unknown | . | Rev |
| ovary | XRN:EBJ7H2 | BRCA1 | SV | likely | FALSE | 843_880>TTACAGCATGAGAACAG | S282_K294>YSMRTE | 0.3354 | chr17 | 41246668 | unknown | homozygous | Rev |
| ovary | XRN:EBJ7H2 | BRCA1 | SV | likely | TRUE | 843_846delCTCA | S282fs*15 | 0.66 | chr17 | 41246701 | germline | homozygous | Sen |
| ovary | XRN:A33RD1 | BRCA1 | SV | likely | TRUE | 3066delA | V1023fs*1 | 0.7788 | chr17 | 41244481 | unknown | homozygous | Sen |
| ovary | XRN:A33RD1 | BRCA1 | SV | likely | FALSE | 2852_3409del558 | R951_P1136del | 0.1869 | chr17 | | unknown | . | Rev |
| ovary | XRN:Q01P4P | BRCA1 | SV | likely | TRUE | 1016_1017insA | V340fs*6 | 0.4052 | chr17 | 41246531 | somatic | homozygous | Sen |
| ovary | XRN:Q01P4P | BRCA1 | SV | likely | TRUE | 953_998del46 | H318fs*8 | 0.2466 | chr17 | | unknown | . | Rev |
| ovary | XRN:A4J2GV | BRCA1 | SV | unknown | FALSE | 4327_4328CG>TC | R1443S | 0.6522 | chr17 | 41234450 | somatic | homozygous | Rev |
| ovary | XRN:A4J2GV | BRCA1 | SV | likely | TRUE | 4327C>T | R1443* | 0.192 | chr17 | 41234451 | germline | not in tumor | Sen |
| ovary | XRN:G77428 | BRCA1 | SV | unknown | FALSE | 5267_5268AG>CA | Q1756P | 0.6674 | chr17 | 41209078 | somatic | homozygous | Rev |
| ovary | XRN:G77428 | BRCA1 | SV | likely | TRUE | 5266_5267insC | Q1756fs*74 | 0.1641 | chr17 | 41209079 | germline | not in tumor | Sen |
| ovary | XRN:C993XM | BRCA1 | SV | likely | FALSE | 4327_4329CGA>TGG | R1443W | 0.3532 | chr17 | 41234449 | unknown | homozygous | Rev |
| ovary | XRN:C993XM | BRCA1 | SV | likely | TRUE | 4327C>T | R1443* | 0.5043 | chr17 | 41234451 | unknown | homozygous | Sen |
| ovary | XRN:C993XM | BRCA1 | SV | unknown | FALSE | 4329A>G | R1443R | 0.3501 | chr17 | 41234449 | unknown | homozygous | Rev |
| ovary | XRN:MRWJE2 | BRCA1 | SV | likely | FALSE | 3045_3701del657 | N1016_V1234del | 0.61 | chr17 | | unknown | . | Rev |
| ovary | XRN:MRWJE2 | BRCA1 | SV | likely | TRUE | 3479_3489delAGGAAGATACT | E1161fs*3 | 0.5 | chr17 | 41244058 | somatic | homozygous | Sen |
| ovary | XRN:SAWD73 | BRCA1 | SV | unknown | FALSE | 1963T>G | Y655D | 0.2475 | chr17 | 41245585 | germline | not in tumor | Rev |
| ovary | XRN:SAWD73 | BRCA1 | SV | known | TRUE | 1961_1962insA | Y655fs*18 | 0.4537 | chr17 | 41245586 | unknown | homozygous | Sen |
| ovary | XRN:QTJ3FW | BRCA1 | SV | likely | TRUE | 4327C>T | R1443* | 0.6038 | chr17 | 41234451 | somatic | homozygous | Sen |
| ovary | XRN:QTJ3FW | BRCA1 | SV | likely | FALSE | 4327_4329CGA>TGT | R1443C | 0.2158 | chr17 | 41234449 | germline | not in tumor | Rev |
| ovary | XRN:QTJ3FW | BRCA1 | SV | unknown | FALSE | 4329A>T | R1443R | 0.2182 | chr17 | 41234449 | germline | not in tumor | Rev |
| ovary | XRN:9PS28K | BRCA1 | SV | likely | TRUE | 5277delG | I1760fs*5 | 0.1186 | chr17 | 41209068 | somatic | homozygous | Rev |
| ovary | XRN:9PS28K | BRCA1 | SV | likely | TRUE | 5266_5267insC | Q1756fs*74 | 0.6902 | chr17 | 41209079 | unknown | homozygous | Sen |
| ovary | XRN:Y8EX6Q | BRCA1 | SV | likely | FALSE | 4183_4184CA>TC | Q1395S | 0.1935 | chr17 | 41242962 | germline | not in tumor | Rev |
| ovary | XRN:Y8EX6Q | BRCA1 | SV | likely | TRUE | 4183C>T | Q1395* | 0.6907 | chr17 | 41242963 | somatic | homozygous | Sen |
| ovary | XRN:PR99F5 | BRCA1 | SV | likely | TRUE | 3874delT | S1292fs*15 | 0.8103 | chr17 | 41243673 | somatic | homozygous | Sen |
| ovary | XRN:PR99F5 | BRCA1 | SV | likely | FALSE | 3874_3928>CTGCTAGCTTGTTTTCTTCACAGTG | S1292_T1310>LLACFLHSA | 0.1615 | chr17 | 41243617 | unknown | homozygous | Rev |
| ovary | XRN:PR99F5 | BRCA1 | SV | unknown | FALSE | 3871_3873TGT>ATG | C1291M | 0.0744 | chr17 | 41243675 | unknown | homozygous | Rev |
| ovary | XRN:T3VFL1 | BRCA1 | SV | likely | FALSE | 4117_4119GAG>TAC | E1373Y | 0.2783 | chr17 | 41243027 | somatic | homozygous | Rev |
| ovary | XRN:T3VFL1 | BRCA1 | SV | likely | TRUE | 4117G>T | E1373* | 0.3616 | chr17 | 41243029 | germline | not in tumor | Sen |
| ovary | XRN:SFL0AP | BRCA1 | SV | likely | TRUE | 3893C>A | S1298* | 0.75 | chr17 | 41243655 | unknown | homozygous | Sen |
| ovary | XRN:SFL0AP | BRCA1 | SV | unknown | FALSE | 3856_3900del45 | S1286_C1300del | 0.25 | chr17 | | unknown | . | Rev |
| ovary | XRN:9PRM1R | BRCA1 | SV | likely | FALSE | 1316_1341>GCTTAATATTA | A439_V447>GLIL | 0.3406 | chr17 | 41246208 | unknown | homozygous | Rev |
| ovary | XRN:9PRM1R | BRCA1 | SV | likely | TRUE | 1315_1316insG | A439fs*5 | 0.476 | chr17 | 41246232 | unknown | homozygous | Sen |
| ovary | XRN:G4XR44 | BRCA1 | SV | likely | TRUE | 1102G>T | E368* | 0.399 | chr17 | 41246446 | germline | not in tumor | Sen |
| ovary | XRN:G4XR44 | BRCA1 | SV | unknown | FALSE | 1102_1104GAA>TAT | E368Y | 0.2228 | chr17 | 41246444 | somatic | homozygous | Rev |
| ovary | XRN:MJMXX6 | BRCA1 | SV | likely | TRUE | 2215A>T | K739* | 0.199 | chr17 | 41245333 | germline | not in tumor | Sen |
| ovary | XRN:MJMXX6 | BRCA1 | SV | likely | FALSE | 2215_2217AAA>TAT | K739Y | 0.4315 | chr17 | 41245331 | unknown | homozygous | Rev |
| ovary | XRN:XJQL4T | BRCA1 | SV | likely | TRUE | 3748G>T | E1250* | 0.4834 | chr17 | 41243800 | unknown | NA | Sen |
| ovary | XRN:XJQL4T | BRCA1 | SV | likely | FALSE | 3748_3750GAG>TAT | E1250Y | 0.2755 | chr17 | 41243798 | unknown | NA | Rev |
| ovary | XRN:46E4J7 | BRCA1 | SV | likely | TRUE | 1787_4096+340del2650 | splice site 1787_4096+340del2650 | 0.535 | chr17 | | unknown | . | Rev |
| ovary | XRN:46E4J7 | BRCA1 | SV | likely | TRUE | 1953_1954insG | K652fs*21 | 0.6616 | chr17 | 41245594 | somatic | homozygous | Sen |
| ovary | XRN:AD95XJ | BRCA1 | SV | likely | TRUE | 2504_2511delATGAAGTT | H835fs*14 | 0.77 | chr17 | 41245036 | somatic | homozygous | Rev |
| ovary | XRN:AD95XJ | BRCA1 | SV | likely | TRUE | 2456_2456delC | D821fs*25 | 0.86 | chr17 | 41245091 | unknown | homozygous | Sen |
| ovary | XRN:8CKQXX | BRCA1 | SV | likely | FALSE | 2910_2952>CATGGACTTTTACAAAACCCATA TCGTATACCACCAC | K970_F984>NMDFYKTHIVYHH | 0.2235 | chr17 | 41244595 | unknown | . | Rev |
| ovary | XRN:8CKQXX | BRCA1 | SV | likely | TRUE | 2910delA | K970fs*30 | 0.6942 | chr17 | 41244637 | somatic | homozygous | Sen |
| ovary | XRN:397H1Y | BRCA1 | SV | likely | FALSE | 955_1009>GTAATGATAGGCGGACTCCCAGC ACAGA | N319_E337>VMIGGLPAQK | 0.5653 | chr17 | 41246531 | unknown | NA | Rev |
| ovary | XRN:397H1Y | BRCA1 | SV | likely | TRUE | 955_982del28 | N319fs*13 | 0.5659 | chr17 | | unknown | . | Sen |
| ovary | XRN:QURPR4 | BRCA1 | SV | likely | FALSE | 739_789>ACACCACTGAGAAGCGTGCAGCTT | N247_G263>TPLRSVQL | 0.4022 | chr17 | 41246807 | unknown | homozygous | Rev |
| ovary | XRN:QURPR4 | BRCA1 | SV | likely | TRUE | 763_788del26 | E255fs*1 | 0.6019 | chr17 | | unknown | . | Sen |
| ovary | XRN:8PNS11 | BRCA1 | SV | likely | FALSE | 4060_4071delAATAATCAAGAA | N1354_E1357del | 0.38 | chr17 | 41243476 | unknown | homozygous | Rev |
| ovary | XRN:8PNS11 | BRCA1 | SV | likely | TRUE | 4065_4068delTCAA | N1355fs*10 | 0.3133 | chr17 | 41243479 | germline | not in tumor | Sen |
| prostate | XRN:KQJ6XE | BRCA1 | SV | likely | TRUE | 3770_3771delAG | E1257fs*9 | 0.2432 | chr17 | 41243776 | somatic | het | Rev |
| prostate | XRN:KQJ6XE | BRCA1 | SV | likely | TRUE | 3756_3759delGTCT | S1253fs*10 | 0.4323 | chr17 | 41243788 | somatic | het | Sen |

| Tissue | Sample | Gene | variant_type | fmi_reportable_status | hrr | variant_cds | variant_pe | variant_af | chrom | pos | somatic_germline_status | zygosity | Reversion |
|---|---|---|---|---|---|---|---|---|---|---|---|---|---|
| bladder | XRN:JF11GK | BRCA2 | SV | likely | TRUE | 3140_3174del35 | I1047fs*8 | 0.2203 | chr13 | | unknown | . | Rev |
| bladder | XRN:JF11GK | BRCA2 | SV | likely | TRUE | 3189_3192delGTCA | S1064fs*12 | 0.7958 | chr13 | 32911680 | somatic | homozygous | Sen |
| breast | XRN:T1489K | BRCA2 | SV | likely | TRUE | 8744_8747CTTA>AATGCA | Y2916fs*12 | 0.57 | chr13 | 32950920 | somatic | homozygous | Rev |
| breast | XRN:T1489K | BRCA2 | SV | likely | TRUE | 8755-1G>T | splice site 8755-1G>T | 0.8 | chr13 | 32953453 | germline | homozygous | Sen |
| breast | XRN:6HJVM5 | BRCA2 | SV | likely | FALSE | 2765_3829>ATCATCATT | F922_N1277>YHHY | 0.5386 | chr13 | 32911256 | somatic | homozygous | Rev |
| breast | XRN:6HJVM5 | BRCA2 | SV | known | TRUE | 2808_2811delACAA | A938fs*21 | 0.576 | chr13 | 32911299 | somatic | homozygous | Sen |
| breast | XRN:4B3E6Y | BRCA2 | SV | likely | FALSE | 5231_5668delGCATGTCTAACAGCTATTCCTA CCATTCTGATGAGGTATATAATGATTCAGGA TATCTCTCAAAAAATAAACTTGATTCTGGTAT TGAGCCAGTATTGAAGAATGTTGAAGATCAA AAAAACACTAGTTTTTCCAAAGTAATATCCAA | S1744_I1889del | 0.16 | chr13 | 32913722 | germline | not in tumor | Rev |

| | | | | | | | | | | | | | |
|---|---|---|---|---|---|---|---|---|---|---|---|---|---|
| | | | | | | TGTAAAAGATGCAAATGCATACCCACAAACT GTAAATGAAGATATTTGCGTTGAGGAACTTG TGACTAGCTCTTCACCCTGCAAAAATAAAAA TGCAGCCATTAAATTGTCCATATCTAATAGTA ATAATTTTGAGGTAGGGCCACCTGCATTTAG GATAGCCAGTGGTAAAATCGTTTGTGTTTCA CATGAAACAATTAAAAAAGTGAAAGACATATT TACAGACAGTTTCAGTAAAGTAATTAAGGAA AACAACGAGAATAAATCAAAAATTTGCCAAA CGAAAATTA | | | | | | | |
| breast | XRN:4B3E6Y | BRCA2 | SV | likely | TRUE | 5542delA | S1848fs*15 | 0.96 | chr13 | 32914033 | germline | homozygous | Sen |
| breast | XRN:BF489D | BRCA2 | SV | likely | FALSE | 6615_6634GAAAACAAATATAGAAGTTT>AA | K2206_C2212>S | 0.2354 | chr13 | 32915107 | germline | not in tumor | Rev |
| breast | XRN:BF489D | BRCA2 | SV | likely | TRUE | 6615_6628delGAAAACAAATATAG | K2206fs*14 | 0.3695 | chr13 | 32915106 | unknown | homozygous | Sen |
| breast | XRN:DY9QKR | BRCA2 | SV | likely | TRUE | 6275_6276delTT | L2092fs*7 | 0.3814 | chr13 | 32914766 | somatic | homozygous | Sen |
| breast | XRN:DY9QKR | BRCA2 | SV | likely | FALSE | 6088_6276>CTGAGCATAGTC | N2030_L2092>LSIV | 0.4042 | chr13 | 32914580 | somatic | homozygous | Rev |
| breast | XRN:UE3KEM | BRCA2 | SV | likely | TRUE | 4131_4132insTGAGGA | T1378* | 0.7131 | chr13 | 32912623 | somatic | homozygous | Sen |
| breast | XRN:UE3KEM | BRCA2 | SV | likely | FALSE | 3817_4170del354 | F1273_L1390del | 0.2999 | chr13 | | unknown | . | Rev |
| breast | XRN:VBMD1L | BRCA2 | SV | likely | FALSE | 728_733delATGATA | N243_D244del | 0.1474 | chr13 | 32905101 | somatic | het | Rev |
| breast | XRN:VBMD1L | BRCA2 | SV | likely | TRUE | 728_729delAT | N243fs*2 | 0.3161 | chr13 | 32905101 | somatic | het | Sen |
| breast | XRN:91VK98 | BRCA2 | SV | known | TRUE | 2808_2811delACAA | A938fs*21 | 0.7764 | chr13 | 32911299 | somatic | homozygous | Sen |
| breast | XRN:91VK98 | BRCA2 | SV | likely | FALSE | 2670_2840del171 | F890_D946del | 0.3698 | chr13 | | unknown | . | Rev |
| breast | XRN:PU569Q | BRCA2 | SV | likely | FALSE | 5946_5967>G | S1982_S1989>R | 0.1921 | chr13 | 32914438 | germline | not in tumor | Rev |
| breast | XRN:PU569Q | BRCA2 | SV | known | TRUE | 5946delT | S1982fs*22 | 0.5444 | chr13 | 32914437 | somatic | homozygous | Sen |
| breast | XRN:NB8RA6 | BRCA2 | SV | likely | TRUE | 5621_5624delTTAA | I1874fs*34 | 0.4409 | chr13 | 32914112 | somatic | homozygous | Sen |
| breast | XRN:NB8RA6 | BRCA2 | SV | likely | FALSE | 5442_6041del600 | T1815_V2014del | 0.4708 | chr13 | | unknown | . | Rev |
| breast | XRN:4G5QQX | BRCA2 | SV | likely | FALSE | 5794_6471del678 | H1932_Q2157del | 0.3529 | chr13 | | unknown | . | Rev |
| breast | XRN:4G5QQX | BRCA2 | SV | likely | TRUE | 5799_5802delCCAA | N1933fs*29 | 0.7229 | chr13 | 32914290 | somatic | homozygous | Sen |
| breast | XRN:LDG2PG | BRCA2 | SV | likely | FALSE | 5680_5682TAC>GAG | Y1894E | 0.3027 | chr13 | 32914172 | unknown | homozygous | Rev |
| breast | XRN:LDG2PG | BRCA2 | SV | likely | TRUE | 5682C>G | Y1894* | 0.467 | chr13 | 32914174 | somatic | homozygous | Sen |
| breast | XRN:M5E422 | BRCA2 | SV | likely | TRUE | 5675_6841+314del1481 | splice site 5675_6841+314del1481 | 0.1673 | chr13 | | unknown | . | Rev |
| breast | XRN:M5E422 | BRCA2 | SV | likely | TRUE | 6618_6630delAACAAATATAGAA | T2207fs*18 | 0.2592 | chr13 | 32915109 | somatic | homozygous | Rev |
| breast | XRN:M5E422 | BRCA2 | SV | likely | TRUE | 5362_5363insT | S1788fs*19 | 0.5659 | chr13 | 32913854 | germline | homozygous | Sen |
| breast | XRN:M5E422 | BRCA2 | SV | likely | TRUE | 5875_5876insTGTA | K1959fs*2 | 0.2318 | chr13 | 32914367 | somatic | homozygous | Rev |
| breast | XRN:CGUH0J | BRCA2 | SV | unknown | FALSE | 2312_2611del300 | L771_I870del | 0.8057 | chr13 | | unknown | . | Rev |
| breast | XRN:CGUH0J | BRCA2 | SV | likely | TRUE | 2385_2386insAA | D796fs*15 | 0.4111 | chr13 | 32910877 | unknown | homozygous | Sen |
| breast | XRN:YS5MEA | BRCA2 | SV | likely | TRUE | 5800_6841+200del1242 | splice site 5800_6841+200del1242 | 0.2916 | chr13 | | unknown | . | Rev |
| breast | XRN:YS5MEA | BRCA2 | SV | likely | TRUE | 6268_6269delCA | H2090fs*1 | 0.4856 | chr13 | 32914759 | somatic | homozygous | Sen |
| breast | XRN:B9MDNQ | BRCA2 | SV | likely | FALSE | 3533_5185del1653 | S1178_D1728del | 0.2925 | chr13 | | unknown | . | Rev |
| breast | XRN:B9MDNQ | BRCA2 | SV | likely | TRUE | 4936_4939delGAAA | E1646fs*23 | 0.6845 | chr13 | 32913427 | somatic | homozygous | Sen |
| breast | XRN:7BR333 | BRCA2 | SV | likely | FALSE | 5038_5241>AATGGT | S1680_N1747>NG | 0.1918 | chr13 | 32913530 | somatic | homozygous | Rev |
| breast | XRN:7BR333 | BRCA2 | SV | unknown | FALSE | 4625_6013del1389 | V1542_E2004del | 0.0691 | chr13 | | unknown | . | Rev |
| breast | XRN:7BR333 | BRCA2 | SV | likely | TRUE | 5130_5133delTGTA | Y1710fs*1 | 0.508 | chr13 | 32913621 | unknown | homozygous | Sen |
| breast | XRN:LK1US2 | BRCA2 | SV | likely | FALSE | 4121_4214>AGGAGGGAAACACTCAGATTAAA GAA | K1374_N1405>RRETLRLKN | 0.47 | chr13 | 32912612 | unknown | homozygous | Rev |
| breast | XRN:LK1US2 | BRCA2 | SV | likely | TRUE | 4121_4121delA | K1374fs*14 | 0.71 | chr13 | 32912612 | somatic | homozygous | Sen |
| breast | XRN:61XAYJ | BRCA2 | SV | unknown | FALSE | 5630_5785del156 | N1877_E1928del | 0.29 | chr13 | | unknown | . | Rev |
| breast | XRN:61XAYJ | BRCA2 | SV | known | TRUE | 5645C>A | S1882* | 0.78 | chr13 | 32914137 | somatic | homozygous | Sen |
| breast | XRN:KS86WH | BRCA2 | SV | likely | TRUE | 5350_5351delAA | N1784fs*2 | 0.2398 | chr13 | 32913836 | unknown | homozygous | Sen |
| breast | XRN:KS86WH | BRCA2 | SV | likely | FALSE | 5333_5346ATGTTGAAGATCAA>TC | N1778_N1784>IKN | 0.1019 | chr13 | 32913825 | somatic | homozygous | Rev |
| breast | XRN:CJMBLW | BRCA2 | SV | likely | FALSE | 2808_2815ACAAGCAA>GCAAC | A938_T939>P | 0.2915 | chr13 | 32911305 | unknown | homozygous | Rev |
| breast | XRN:CJMBLW | BRCA2 | SV | known | TRUE | 2808_2811delACAA | A938fs*21 | 0.5339 | chr13 | 32911299 | somatic | homozygous | Sen |
| breast | XRN:41A01U | BRCA2 | SV | unknown | FALSE | 6486_6506del21 | K2162_T2169>N | 0.6236 | chr13 | | unknown | . | Rev |
| breast | XRN:41A01U | BRCA2 | SV | likely | TRUE | 6481_6484delGACA | D2161fs*6 | 0.193 | chr13 | 32914972 | germline | not in tumor | Sen |
| breast | XRN:Y9HEXR | BRCA2 | SV | likely | TRUE | 5682C>G | Y1894* | 0.5882 | chr13 | 32914174 | germline | homozygous | Sen |
| breast | XRN:Y9HEXR | BRCA2 | SV | likely | FALSE | 5089_6171del1083 | I1697_G2057del | 0.149 | chr13 | | unknown | . | Rev |
| breast | XRN:CPG8LV | BRCA2 | SV | likely | TRUE | 5146_5149delTATG | Y1716fs*8 | 0.7192 | chr13 | 32913637 | germline | homozygous | Sen |
| breast | XRN:CPG8LV | BRCA2 | SV | likely | FALSE | 4695_5648del954 | T1566_K1883del | 0.0677 | chr13 | | unknown | . | Rev |
| breast | XRN:TUCB63 | BRCA2 | SV | likely | FALSE | 1799_1800AT>TA | Y600L | 0.4219 | chr13 | 32907414 | somatic | homozygous | Rev |
| breast | XRN:TUCB63 | BRCA2 | SV | likely | TRUE | 1800T>A | Y600* | 0.2857 | chr13 | 32907415 | germline | not in tumor | Sen |
| breast | XRN:NPWHFC | BRCA2 | SV | likely | TRUE | 8869C>T | Q2957* | 0.4444 | chr13 | 32953568 | unknown | homozygous | Sen |
| breast | XRN:NPWHFC | BRCA2 | SV | unknown | FALSE | 8872_8880delAAGGAACAA | K2958_Q2960del | 0.4676 | chr13 | 32953570 | unknown | homozygous | Rev |
| breast | XRN:7BNDH6 | BRCA2 | SV | likely | FALSE | 3265_3289>TCAGATGTTA | Q1089_F1097>SDVI | 0.2035 | chr13 | 32911757 | germline | not in tumor | Rev |
| breast | XRN:7BNDH6 | BRCA2 | SV | likely | FALSE | 3265_3296>TCAGATGTTATTTTCCAAGCAGG | Q1089_S1099>SDVIFQAG | 0.1301 | chr13 | 32911757 | germline | not in tumor | Rev |
| breast | XRN:7BNDH6 | BRCA2 | SV | likely | TRUE | 3264_3265insT | Q1089fs*10 | 0.4522 | chr13 | 32911756 | unknown | homozygous | Sen |
| breast | XRN:ARDKJ9 | BRCA2 | SV | likely | TRUE | 6491delA | Q2164fs*4 | 0.9282 | chr13 | 32914982 | germline | homozygous | Sen |
| breast | XRN:ARDKJ9 | BRCA2 | SV | likely | TRUE | 6472_6485delTTTCAACAAGACAA | F2158fs*13 | 0.5008 | chr13 | 32914963 | unknown | homozygous | Rev |
| breast | XRN:96QE4H | BRCA2 | SV | likely | FALSE | 5812_5841del30 | G1938_P1947del | 0.1632 | chr13 | | unknown | . | Rev |
| breast | XRN:96QE4H | BRCA2 | SV | likely | TRUE | 5785_5798delATTTTACAACATAA | I1929fs*11 | 0.1187 | chr13 | 32914276 | unknown | homozygous | Rev |
| breast | XRN:96QE4H | BRCA2 | SV | likely | TRUE | 5828delC | S1943fs*20 | 0.8227 | chr13 | 32914319 | unknown | homozygous | Sen |
| breast | XRN:YUARFF | BRCA2 | SV | likely | TRUE | 1796_1800delCTTAT | S599fs*1 | 0.3781 | chr13 | 32907410 | unknown | homozygous | Sen |
| breast | XRN:YUARFF | BRCA2 | SV | likely | FALSE | 1789_1798GAAACATCTT>AAAC | E597_Y600>KH | 0.2637 | chr13 | 32907404 | unknown | homozygous | Rev |

| | | | | | | | | | | | | | |
|---|---|---|---|---|---|---|---|---|---|---|---|---|---|
| breast | XRN:14K7MQ | BRCA2 | SV | likely | TRUE | 5616_5620delAGTAA | K1872fs*2 | 0.7917 | chr13 | 32914107 | germline | homozygous | Sen |
| breast | XRN:14K7MQ | BRCA2 | SV | likely | FALSE | 5336_6160del825 | V1779_S2053del | 0.3015 | chr13 | | unknown | . | Rev |
| breast | XRN:NETHD6 | BRCA2 | SV | likely | TRUE | 2957_2958insG | N986fs*2 | 0.7602 | chr13 | 32911449 | somatic | homozygous | Sen |
| breast | XRN:NETHD6 | BRCA2 | SV | likely | FALSE | 2957_2958insAAG | N986>KS | 0.1073 | chr13 | 32911449 | germline | not in tumor | Rev |
| breast | XRN:CM7DU4 | BRCA2 | SV | likely | TRUE | 2380_2381insA | M794fs*8 | 0.3514 | chr13 | 32910872 | somatic | het | Sen |
| breast | XRN:CM7DU4 | BRCA2 | SV | likely | FALSE | 2358_2384del27 | R787_S795del | 0.0605 | chr13 | | unknown | . | Rev |
| breast | XRN:V30G6V | BRCA2 | SV | likely | FALSE | 755_771ACAGTGAAAACACAAAT>TGAAAACA | D252_N257>VKT | 0.0647 | chr13 | 32905129 | germline | not in tumor | Rev |
| breast | XRN:V30G6V | BRCA2 | SV | likely | TRUE | 755_758delACAG | D252fs*24 | 0.1529 | chr13 | 32905128 | germline | not in tumor | Sen |
| breast | XRN:V30G6V | BRCA2 | SV | likely | FALSE | 755_765ACAGTGAAAAC>TGAAA | D252_N255>VK | 0.1353 | chr13 | 32905129 | germline | not in tumor | Rev |
| breast | XRN:V30G6V | BRCA2 | SV | likely | TRUE | 802_842del41 | K268fs*13 | 0.0938 | chr13 | | unknown | . | Rev |
| breast | XRN:RFRU6E | BRCA2 | SV | likely | TRUE | 5576_5579delTTAA | I1859fs*3 | 0.5535 | chr13 | 32914067 | unknown | het | Sen |
| breast | XRN:RFRU6E | BRCA2 | SV | likely | FALSE | 4267_5685del1419 | T1423_E1895del | 0.1011 | chr13 | | unknown | . | Rev |
| breast | XRN:311QN1 | BRCA2 | SV | likely | TRUE | 6405_6409delCTTAA | N2135fs*3 | 0.6773 | chr13 | 32914896 | unknown | homozygous | Sen |
| breast | XRN:311QN1 | BRCA2 | SV | likely | FALSE | 6360_6443del84 | E2121_S2148del | 0.1694 | chr13 | | unknown | . | Rev |
| breast | XRN:1908J2 | BRCA2 | SV | likely | FALSE | 7480_7481CG>TC | R2494S | 0.398 | chr13 | 32930609 | somatic | homozygous | Rev |
| breast | XRN:1908J2 | BRCA2 | SV | likely | TRUE | 7480C>T | R2494* | 0.2901 | chr13 | 32930609 | germline | not in tumor | Sen |
| breast | XRN:PVB7E9 | BRCA2 | SV | likely | FALSE | 4763_6250del1488 | A1588_F2083del | 0.4852 | chr13 | | unknown | . | Rev |
| breast | XRN:PVB7E9 | BRCA2 | SV | likely | TRUE | 5682C>G | Y1894* | 0.4973 | chr13 | 32914174 | somatic | homozygous | Sen |
| breast | XRN:NMXSWW | BRCA2 | SV | likely | FALSE | 810_1025>GGGAATTCATTT | N272_E342>IHL | 0.3124 | chr13 | 32906425 | unknown | homozygous | Rev |
| breast | XRN:NMXSWW | BRCA2 | SV | likely | TRUE | 810delA | N272fs*5 | 0.8421 | chr13 | 32906424 | unknown | homozygous | Sen |
| breast | XRN:61RW6R | BRCA2 | SV | likely | TRUE | 3922G>T | E1308* | 0.66 | chr13 | 32912414 | unknown | homozygous | Sen |
| breast | XRN:61RW6R | BRCA2 | SV | unknown | FALSE | 3924A>C | E1308D | 0.15 | chr13 | 32912416 | unknown | homozygous | Rev |
| breast | XRN:SN2SFF | BRCA2 | SV | likely | TRUE | 655_681+333del360 | splice site 655_681+333del360 | 0.2425 | chr13 | | unknown | . | Rev |
| breast | XRN:SN2SFF | BRCA2 | SV | likely | TRUE | 631+1G>A | splice site 631+1G>A | 0.7477 | chr13 | 32900751 | germline | homozygous | Sen |
| breast | XRN:NWYRKQ | BRCA2 | SV | likely | TRUE | 1929delG | R645fs*15 | 0.5933 | chr13 | 32910420 | germline | homozygous | Sen |
| breast | XRN:NWYRKQ | BRCA2 | SV | likely | TRUE | 1941_1957delTTCACAGAATGATTCTG | C647fs*1 | 0.2293 | chr13 | 32910432 | somatic | homozygous | Rev |
| breast | XRN:QTWLGH | BRCA2 | SV | likely | FALSE | 3860_3916>TAATAA | N1287_V1306>III | 0.2588 | chr13 | 32912352 | unknown | homozygous | Rev |
| breast | XRN:QTWLGH | BRCA2 | SV | likely | TRUE | 3860delA | N1287fs*6 | 0.5435 | chr13 | 32912351 | unknown | homozygous | Sen |
| breast | XRN:TV5RE9 | BRCA2 | SV | likely | FALSE | 2205_3155del951 | A736_A1052del | 0.1366 | chr13 | | unknown | . | Rev |
| breast | XRN:TV5RE9 | BRCA2 | SV | likely | TRUE | 2204_2205insC | A736fs*15 | 0.4057 | chr13 | 32910696 | germline | not in tumor | Sen |
| breast | XRN:2P9XL4 | BRCA2 | SV | likely | TRUE | 5576_5579delTTAA | I1859fs*3 | 0.2867 | chr13 | 32914067 | germline | not in tumor | Sen |
| breast | XRN:2P9XL4 | BRCA2 | SV | likely | FALSE | 5571_5603del33 | E1857_T1867del | 0.437 | chr13 | | unknown | . | Rev |
| lung | XRN:TKS8GL | BRCA2 | SV | likely | TRUE | 2426T>G | L809* | 0.5074 | chr13 | 32910918 | unknown | homozygous | Sen |
| lung | XRN:TKS8GL | BRCA2 | SV | unknown | FALSE | 2426_2427TA>GG | L809W | 0.2944 | chr13 | 32910918 | unknown | homozygous | Rev |
| lung | XRN:B3ND03 | BRCA2 | SV | likely | TRUE | 1789delG | E597fs*17 | 0.259 | chr13 | 32907403 | unknown | homozygous | Sen |
| lung | XRN:B3ND03 | BRCA2 | SV | likely | FALSE | 1789_1814>AAACATCTTATAAAGGAAAAAAAAT | E597_I605>KHLIKEKKI | 0.2449 | chr13 | 32907428 | unknown | homozygous | Rev |
| lung | XRN:M434KS | BRCA2 | SV | likely | TRUE | 1817_1819CGA>TTT | P606_K607>L* | 0.4735 | chr13 | 32907432 | germline | het | Sen |
| lung | XRN:M434KS | BRCA2 | SV | unknown | FALSE | 1818G>T | P606P | 0.47 | chr13 | 32907433 | germline | het | Rev |
| lung | XRN:5FUJHA | BRCA2 | SV | likely | TRUE | 6110_6110delA | E2037fs*3 | 0.25 | chr13 | 32914601 | unknown | homozygous | Sen |
| lung | XRN:5FUJHA | BRCA2 | SV | unknown | FALSE | 6082_6111delGAAGAAAATACTGCTATACGTA CTCCAGAA | E2028_E2037del | 0.76 | chr13 | 32914573 | somatic | homozygous | Rev |
| ovary | XRN:TVKR6E | BRCA2 | SV | unknown | FALSE | 2470_3207del738 | L824_S1069del | 0.279 | chr13 | | unknown | . | Rev |
| ovary | XRN:TVKR6E | BRCA2 | SV | likely | TRUE | 2957_2958insA | N986fs*2 | 0.5336 | chr13 | 32911449 | somatic | homozygous | Sen |
| ovary | XRN:3GQ05E | BRCA2 | SV | known | TRUE | 5946delT | S1982fs*22 | 0.8923 | chr13 | 32914437 | somatic | homozygous | Sen |
| ovary | XRN:3GQ05E | BRCA2 | SV | likely | FALSE | 5385_6152del768 | K1795_V2050del | 0.2567 | chr13 | | unknown | . | Rev |
| ovary | XRN:990QLG | BRCA2 | SV | likely | FALSE | 4613_4633del21 | S1538_L1545>F | 0.1766 | chr13 | | unknown | . | Rev |
| ovary | XRN:990QLG | BRCA2 | SV | likely | TRUE | 4631delA | N1544fs*24 | 0.6552 | chr13 | 32913122 | unknown | homozygous | Sen |
| ovary | XRN:H474MF | BRCA2 | SV | unknown | FALSE | 2015_2032delGAAATGAAACATGTTCTA | R672_S677del | 0.7969 | chr13 | 32910506 | unknown | homozygous | Rev |
| ovary | XRN:H474MF | BRCA2 | SV | likely | TRUE | 2020G>T | E674* | 0.3333 | chr13 | 32910512 | unknown | homozygous | Sen |
| ovary | XRN:9DGL41 | BRCA2 | SV | likely | FALSE | 4792_6597del1806 | L1598_T2199del | 0.25 | chr13 | | unknown | . | Rev |
| ovary | XRN:9DGL41 | BRCA2 | SV | likely | TRUE | 5130_5133delTGTA | Y1710fs*1 | 0.6 | chr13 | 32913621 | somatic | homozygous | Sen |
| ovary | XRN:GDTE3M | BRCA2 | SV | likely | FALSE | 4174_4542del369 | V1392_E1514del | 0.2087 | chr13 | | unknown | . | Rev |
| ovary | XRN:GDTE3M | BRCA2 | SV | likely | TRUE | 4444G>T | E1482* | 0.2041 | chr13 | 32912936 | unknown | homozygous | Sen |
| ovary | XRN:6UN9V2 | BRCA2 | SV | likely | TRUE | 5864C>A | S1955* | 0.2256 | chr13 | 32914356 | unknown | homozygous | Sen |
| ovary | XRN:6UN9V2 | BRCA2 | SV | likely | FALSE | 5863_5864TC>GA | S1955E | 0.0526 | chr13 | 32914355 | somatic | homozygous | Rev |
| ovary | XRN:6UN9V2 | BRCA2 | SV | likely | FALSE | 5848_5868del21 | V1950_D1956del | 0.0777 | chr13 | | unknown | . | Rev |
| ovary | XRN:6UN9V2 | BRCA2 | SV | likely | FALSE | 5730_6056del327 | D1911_N2019del | 0.016 | chr13 | | unknown | . | Rev |
| ovary | XRN:KUBX58 | BRCA2 | SV | likely | FALSE | 3826_3848>AAAAC | E1276_V1283>KL | 0.05 | chr13 | 32912317 | somatic | homozygous | Rev |
| ovary | XRN:KUBX58 | BRCA2 | SV | likely | TRUE | 3847_3848delGT | V1283fs*2 | 0.63 | chr13 | 32912338 | somatic | homozygous | Sen |
| ovary | XRN:V9U0M8 | BRCA2 | SV | likely | TRUE | 4284_4285insT | Q1429fs*9 | 0.6799 | chr13 | 32912776 | somatic | homozygous | Sen |
| ovary | XRN:V9U0M8 | BRCA2 | SV | likely | FALSE | 4264_4281delGAGACTTCTGATACATTT | E1422_F1427del | 0.089 | chr13 | 32912755 | unknown | homozygous | Rev |
| ovary | XRN:KYV121 | BRCA2 | SV | likely | FALSE | 4478_4505>TGTCCCA | E1493_Q1502>VSQ | 0.1099 | chr13 | 32912980 | germline | not in tumor | Rev |
| ovary | XRN:KYV121 | BRCA2 | SV | likely | FALSE | 4431_4916del486 | L1478_V1639del | 0.0547 | chr13 | | unknown | . | Rev |
| ovary | XRN:KYV121 | BRCA2 | SV | likely | TRUE | 4478_4481delAAAG | E1493fs*10 | 0.7374 | chr13 | 32912969 | somatic | homozygous | Sen |
| ovary | XRN:KYV121 | BRCA2 | SV | likely | FALSE | 4452_4541del90 | D1484_D1513del | 0.1569 | chr13 | | unknown | . | Rev |
| ovary | XRN:KYV121 | BRCA2 | SV | likely | FALSE | 4340_4639del300 | V1447_F1546del | 0.1109 | chr13 | | unknown | . | Rev |
| ovary | XRN:8K2FY3 | BRCA2 | SV | known | TRUE | 5946delT | S1982fs*22 | 0.4189 | chr13 | 32914437 | somatic | homozygous | Sen |
| ovary | XRN:8K2FY3 | BRCA2 | SV | likely | FALSE | 5203_6012del810 | K1735_E2004del | 0.1378 | chr13 | | unknown | . | Rev |
| ovary | XRN:8K2FY3 | BRCA2 | SV | likely | FALSE | 5923_5955del33 | C1975_S1985del | 0.1645 | chr13 | | unknown | . | Rev |
| ovary | XRN:28FVRL | BRCA2 | SV | likely | FALSE | 3226_5796del2571 | V1076_H1932del | 0.3921 | chr13 | | unknown | . | Rev |

| | | | | | | | | | | | | | |
|---|---|---|---|---|---|---|---|---|---|---|---|---|---|
| ovary | XRN:28FVRL | BRCA2 | SV | likely | TRUE | 3978_3979insTGCT | A1327fs*4 | 0.7105 | chr13 | 32912470 | unknown | homozygous | Sen |
| ovary | XRN:YY3F11 | BRCA2 | SV | likely | TRUE | 6468_6469delTC | Q2157fs*18 | 0.38 | chr13 | 32914959 | unknown | homozygous | Sen |
| ovary | XRN:YY3F11 | BRCA2 | SV | likely | FALSE | 6468_6523>TCAATTTCAACAAGACAAACAAC AGTTGAG | S2156_E2175>SISTRQTTVE | 0.3794 | chr13 | 32914987 | unknown | homozygous | Rev |
| ovary | XRN:W6ASDD | BRCA2 | SV | likely | TRUE | 3860_3861insA | N1287fs*2 | 0.4347 | chr13 | 32912352 | unknown | homozygous | Sen |
| ovary | XRN:W6ASDD | BRCA2 | SV | unknown | FALSE | 3860_3861insAAA | K1286_N1287insK | 0.1505 | chr13 | 32912352 | unknown | homozygous | Rev |
| ovary | XRN:VYPUT0 | BRCA2 | SV | likely | FALSE | 4750_6090del1341 | E1584_N2030del | 0.1788 | chr13 | | unknown | . | Rev |
| ovary | XRN:VYPUT0 | BRCA2 | SV | likely | TRUE | 5909C>A | S1970* | 0.9369 | chr13 | 32914401 | somatic | homozygous | Sen |
| ovary | XRN:TM97UU | BRCA2 | SV | likely | FALSE | 6130_6135delGGCTTT | G2044_F2045del | 0.1335 | chr13 | 32914621 | unknown | homozygous | Rev |
| ovary | XRN:TM97UU | BRCA2 | SV | likely | TRUE | 6129_6130insA | G2044fs*5 | 0.5556 | chr13 | 32914621 | somatic | homozygous | Sen |
| ovary | XRN:338WJ3 | BRCA2 | SV | likely | TRUE | 5350_5351delAA | N1784fs*2 | 0.6436 | chr13 | 32913841 | somatic | homozygous | Sen |
| ovary | XRN:338WJ3 | BRCA2 | SV | likely | FALSE | 5209_5658del450 | D1737_Q1886del | 0.2597 | chr13 | | unknown | . | Rev |
| ovary | XRN:LWTH5S | BRCA2 | SV | unknown | FALSE | 4957_5397del441 | T1653_A1799del | 0.2326 | chr13 | | unknown | . | Rev |
| ovary | XRN:LWTH5S | BRCA2 | SV | likely | TRUE | 4965C>G | Y1655* | 0.6522 | chr13 | 32913457 | somatic | homozygous | Sen |
| ovary | XRN:9MGTU2 | BRCA2 | SV | likely | TRUE | 4325C>A | S1442* | 0.6096 | chr13 | 32912817 | germline | homozygous | Sen |
| ovary | XRN:9MGTU2 | BRCA2 | SV | unknown | FALSE | 4298_4339del42 | G1433_I1446del | 0.1528 | chr13 | | unknown | . | Rev |
| ovary | XRN:SJFWA3 | BRCA2 | SV | likely | TRUE | 5576_5579delTTAA | I1859fs*3 | 0.1555 | chr13 | 32914067 | germline | not in tumor | Sen |
| ovary | XRN:SJFWA3 | BRCA2 | SV | likely | FALSE | 5576_5581delTTAAAA | I1859_K1860del | 0.6502 | chr13 | 32914067 | somatic | homozygous | Rev |
| ovary | XRN:LDEQCQ | BRCA2 | SV | likely | FALSE | 5946_5948TGG>GGA | S1982_G1983>RE | 0.3088 | chr13 | 32914438 | unknown | homozygous | Rev |
| ovary | XRN:LDEQCQ | BRCA2 | SV | known | TRUE | 5946delT | S1982fs*22 | 0.5167 | chr13 | 32914437 | unknown | homozygous | Sen |
| ovary | XRN:LDEQCQ | BRCA2 | SV | likely | FALSE | 5942_5968del27 | A1981_S1989del | 0.2467 | chr13 | | unknown | . | Rev |
| ovary | XRN:A45J0L | BRCA2 | SV | likely | FALSE | 5737_6717del981 | C1913_E2239del | 0.2396 | chr13 | | unknown | . | Rev |
| ovary | XRN:A45J0L | BRCA2 | SV | likely | TRUE | 6037A>T | K2013* | 0.4764 | chr13 | 32914529 | unknown | homozygous | Sen |
| ovary | XRN:YU87W1 | BRCA2 | SV | likely | FALSE | 3630_4451del822 | E1211_D1484del | 0.4253 | chr13 | | unknown | . | Rev |
| ovary | XRN:YU87W1 | BRCA2 | SV | likely | FALSE | 3832_3861del30 | H1278_N1287del | 0.2111 | chr13 | | unknown | . | Rev |
| ovary | XRN:YU87W1 | BRCA2 | SV | likely | TRUE | 3847_3848delGT | V1283fs*2 | 0.3862 | chr13 | 32912338 | unknown | homozygous | Sen |
| ovary | XRN:5DM7DW | BRCA2 | SV | likely | TRUE | 2151delT | C717fs*13 | 0.272 | chr13 | 32910642 | unknown | homozygous | Rev |
| ovary | XRN:5DM7DW | BRCA2 | SV | likely | TRUE | 2235_2235delA | V746fs*26 | 0.1106 | chr13 | 32910726 | germline | not in tumor | Rev |
| ovary | XRN:5DM7DW | BRCA2 | SV | likely | TRUE | 2175_2176insA | V726fs*25 | 0.7204 | chr13 | 32910667 | somatic | homozygous | Sen |
| ovary | XRN:46J8EX | BRCA2 | SV | likely | FALSE | 4936_6233>CAGCAAAA | E1646_G2078>QQK | 0.6525 | chr13 | 32913428 | somatic | homozygous | Rev |
| ovary | XRN:46J8EX | BRCA2 | SV | likely | TRUE | 4936_4939delGAAA | E1646fs*23 | 0.8241 | chr13 | 32913427 | germline | homozygous | Sen |
| ovary | XRN:3X3GEJ | BRCA2 | SV | likely | TRUE | 7412delC | T2471fs*8 | 0.1267 | chr13 | 32929401 | germline | not in tumor | Sen |
| ovary | XRN:3X3GEJ | BRCA2 | SV | likely | FALSE | 7412_7430CAAAGTGTGAAGAAGAACC>AAAG | T2471_P2477>KS | 0.1242 | chr13 | 32929402 | germline | not in tumor | Rev |
| ovary | XRN:A3YSAK | BRCA2 | SV | likely | TRUE | 3847_3848delGT | V1283fs*2 | 0.4836 | chr13 | 32912338 | somatic | homozygous | Sen |
| ovary | XRN:A3YSAK | BRCA2 | SV | likely | FALSE | 3721_4443del723 | F1241_E1481del | 0.6704 | chr13 | | unknown | . | Rev |
| ovary | XRN:J1DHF8 | BRCA2 | SV | likely | FALSE | 8297_8309CACCTCTTGAAGC>A | T2766_A2770>N | 0.5103 | chr13 | 32937636 | somatic | homozygous | Rev |
| ovary | XRN:J1DHF8 | BRCA2 | SV | likely | TRUE | 8297delC | T2766fs*11 | 0.1013 | chr13 | 32937635 | unknown | homozygous | Sen |
| ovary | XRN:S6JGEV | BRCA2 | SV | known | TRUE | 5946delT | S1982fs*22 | 0.4619 | chr13 | 32914437 | unknown | homozygous | Sen |
| ovary | XRN:S6JGEV | BRCA2 | SV | likely | TRUE | 5818_6841+277del1301 | splice site 5818_6841+277del1301 | 0.5469 | chr13 | | unknown | . | Rev |
| ovary | XRN:TEH47M | BRCA2 | SV | likely | FALSE | 5649_5679>CGAAAATTATGGCAGGTTGTTAAC | K1883_Y1893>RKLWQVVN | 0.12 | chr13 | 32914141 | unknown | homozygous | Rev |
| ovary | XRN:TEH47M | BRCA2 | SV | likely | TRUE | 5681_5682insA | Y1894fs*1 | 0.63 | chr13 | 32914173 | somatic | homozygous | Sen |
| ovary | XRN:AQP6N4 | BRCA2 | SV | likely | TRUE | 6448_6449insTA | K2150fs*19 | 0.3825 | chr13 | 32914940 | somatic | homozygous | Sen |
| ovary | XRN:AQP6N4 | BRCA2 | SV | likely | FALSE | 6427_6456del30 | S2143_S2152del | 0.0785 | chr13 | | unknown | . | Rev |
| ovary | XRN:AQP6N4 | BRCA2 | SV | likely | FALSE | 6449_6471>TAAAG | K2150_Q2157>IK | 0.1997 | chr13 | 32914943 | unknown | homozygous | Rev |
| ovary | XRN:KGXFFC | BRCA2 | SV | likely | TRUE | 4638delT | F1546fs*22 | 0.59 | chr13 | 32913129 | unknown | homozygous | Sen |
| ovary | XRN:KGXFFC | BRCA2 | SV | likely | FALSE | 4638_4672>GATGAAAAAGAGCA | F1546_S1558>LMKKSS | 0.26 | chr13 | 32913144 | unknown | homozygous | Rev |
| ovary | XRN:7JMD9B | BRCA2 | SV | likely | TRUE | 6136delT | S2046fs*5 | 0.13 | chr13 | 32914627 | somatic | homozygous | Sen |
| ovary | XRN:7JMD9B | BRCA2 | SV | likely | FALSE | 6129_6152delAGGCTTTTCATATAATGTGGTAAA | K2043_V2050del | 0.12 | chr13 | 32914620 | somatic | homozygous | Rev |
| ovary | XRN:7JMD9B | BRCA2 | SV | unknown | FALSE | 6105_6106insGCTATACGTACT | T2035_P2036insAIRT | 0.09 | chr13 | 32914597 | somatic | homozygous | Rev |
| ovary | XRN:7JMD9B | BRCA2 | SV | likely | FALSE | 6136_6155>CATATAATGTGGTATAT | S2046_S2052>HIMYI | 0.13 | chr13 | 32914627 | somatic | homozygous | Rev |
| ovary | XRN:L1RHM7 | BRCA2 | SV | likely | FALSE | 2818_2820CAA>TAT | Q940Y | 0.4368 | chr13 | 32911310 | somatic | homozygous | Rev |
| ovary | XRN:L1RHM7 | BRCA2 | SV | likely | TRUE | 2818C>T | Q940* | 0.2788 | chr13 | 32911310 | somatic | homozygous | Sen |
| ovary | XRN:YUNK0W | BRCA2 | SV | known | TRUE | 5946delT | S1982fs*22 | 0.378 | chr13 | 32914437 | unknown | homozygous | Sen |
| ovary | XRN:YUNK0W | BRCA2 | SV | unknown | FALSE | 5908_5958del51 | S1970_V1986del | 0.562 | chr13 | | unknown | . | Rev |
| pancreas | XRN:903H28 | BRCA2 | SV | likely | TRUE | 5966C>G | S1989* | 0.4327 | chr13 | 32914458 | unknown | homozygous | Sen |
| pancreas | XRN:903H28 | BRCA2 | SV | unknown | FALSE | 5956_5973delGTCCAGGTATCAGATGCT | V1986_A1991del | 0.0895 | chr13 | 32914447 | somatic | homozygous | Rev |
| pancreas | XRN:P3ATLW | BRCA2 | SV | known | TRUE | 5946delT | S1982fs*22 | 0.5014 | chr13 | 32914437 | somatic | homozygous | Sen |
| pancreas | XRN:P3ATLW | BRCA2 | SV | likely | FALSE | 5946_5988>GGAAAATCTGTCC | S1982_A1996>RENLS | 0.2057 | chr13 | 32914453 | unknown | homozygous | Rev |
| pancreas | XRN:RHNBLF | BRCA2 | SV | likely | FALSE | 5465_5922>TCCCACCC | N1822_T1974>IPP | 0.2452 | chr13 | 32913982 | unknown | homozygous | Rev |
| pancreas | XRN:RHNBLF | BRCA2 | SV | likely | TRUE | 5465_5487del23 | N1822fs*4 | 0.3455 | chr13 | | unknown | . | Rev |
| pancreas | XRN:RHNBLF | BRCA2 | SV | likely | TRUE | 5851_5854delAGTT | S1951fs*11 | 0.4505 | chr13 | 32914342 | unknown | homozygous | Sen |
| pancreas | XRN:XUTG0A | BRCA2 | SV | likely | FALSE | 3170_3337>CTGAGC | K1057_E1113>TEQ | 0.846 | chr13 | 32911662 | unknown | homozygous | Rev |
| pancreas | XRN:XUTG0A | BRCA2 | SV | likely | TRUE | 3170_3174delAGAAA | K1057fs*8 | 0.071 | chr13 | 32911661 | unknown | homozygous | Sen |
| pancreas | XRN:RKU9TD | BRCA2 | SV | likely | FALSE | 6380_6490>30 | S2127_Q2164>ISLSISTRQTK | 0.14 | chr13 | 32914871 | unknown | homozygous | Rev |
| pancreas | XRN:RKU9TD | BRCA2 | SV | likely | TRUE | 6490_6490delC | Q2164fs*4 | 0.58 | chr13 | 32914981 | somatic | homozygous | Sen |
| pancreas | XRN:5LVJ54 | BRCA2 | SV | likely | TRUE | 2830A>T | K944* | 0.8904 | chr13 | 32911322 | unknown | homozygous | Sen |
| pancreas | XRN:5LVJ54 | BRCA2 | SV | likely | FALSE | 2173_3096del924 | K725_K1032del | 0.2247 | chr13 | | unknown | . | Rev |
| prostate | XRN:2Y9BY0 | BRCA2 | SV | likely | FALSE | 9382_9384CGA>TGG | R3128W | 0.794 | chr13 | 32968951 | somatic | homozygous | Rev |
| prostate | XRN:2Y9BY0 | BRCA2 | SV | likely | TRUE | 9382C>T | R3128* | 0.103 | chr13 | 32968951 | germline | not in tumor | Sen |
| prostate | XRN:2Y9BY0 | BRCA2 | SV | unknown | FALSE | 9384A>G | R3128R | 0.7881 | chr13 | 32968953 | somatic | homozygous | Rev |

| | | | | | | | | | | | | | |
|---|---|---|---|---|---|---|---|---|---|---|---|---|---|
| prostate | XRN:7DT78E | BRCA2 | SV | known | TRUE | 5946delT | S1982fs*22 | 0.833 | chr13 | 32914437 | germline | homozygous | Sen |
| prostate | XRN:7DT78E | BRCA2 | SV | likely | FALSE | 5259_6215del957 | D1754_S2072del | 0.3711 | chr13 | | unknown | . | Rev |
| prostate | XRN:Y8S3DF | BRCA2 | SV | likely | TRUE | 3847_3848delGT | V1283fs*2 | 0.3546 | chr13 | 32912338 | somatic | het | Sen |
| prostate | XRN:Y8S3DF | BRCA2 | SV | likely | FALSE | 3817_3847>ATAAAAC | F1273_V1283>IKL | 0.3782 | chr13 | 32912308 | somatic | het | Rev |
| prostate | XRN:KCPJQK | BRCA2 | SV | likely | FALSE | 5843_6064del222 | C1948_H2021del | 0.1256 | chr13 | | unknown | . | Rev |
| prostate | XRN:KCPJQK | BRCA2 | SV | likely | FALSE | 5863_5864TC>GA | S1955E | 0.1274 | chr13 | 32914355 | somatic | het | Rev |
| prostate | XRN:KCPJQK | BRCA2 | SV | likely | FALSE | 5864_5865CA>AT | S1955Y | 0.15 | chr13 | 32914356 | somatic | het | Rev |
| prostate | XRN:KCPJQK | BRCA2 | SV | likely | TRUE | 5864C>A | S1955* | 0.1155 | chr13 | 32914356 | somatic | het | Sen |
| prostate | XRN:5BG74Q | BRCA2 | SV | likely | TRUE | 6308C>A | S2103* | 0.64 | chr13 | 32914800 | unknown | homozygous | Sen |
| prostate | XRN:5BG74Q | BRCA2 | SV | unknown | FALSE | 6308CA>AC | S2103Y | 0.2 | chr13 | 32914800 | unknown | homozygous | Rev |
| prostate | XRN:5CNANX | BRCA2 | SV | likely | FALSE | 752_779>AAACACAAATCAAAGA | T251_E260>KTQIKE | 0.74 | chr13 | 32905125 | somatic | homozygous | Rev |
| prostate | XRN:5CNANX | BRCA2 | SV | likely | TRUE | 778_779delGA | E260fs*15 | 0.04 | chr13 | 32905151 | somatic | homozygous | Sen |
| prostate | XRN:C38R2M | BRCA2 | SV | likely | TRUE | 2330_2331insA | D777fs*11 | 0.2466 | chr13 | 32910822 | unknown | homozygous | Sen |
| prostate | XRN:C38R2M | BRCA2 | SV | likely | FALSE | 2331_2346TGTTCTGTCAAACCTA>ATGTTCT | D777_L782>ECS | 0.1344 | chr13 | 32910823 | somatic | homozygous | Rev |
| prostate | XRN:5MWY46 | BRCA2 | SV | known | TRUE | 5946delT | S1982fs*22 | 0.57 | chr13 | 32914437 | somatic | homozygous | Sen |
| prostate | XRN:5MWY46 | BRCA2 | SV | unknown | FALSE | 5632_5985del354 | N1878_N1995del | 0.19 | chr13 | | unknown | . | Rev |
| prostate | XRN:5MWY46 | BRCA2 | SV | unknown | FALSE | 5644_6732del1089 | S1882_K2244del | 0.29 | chr13 | | unknown | . | Rev |
| prostate | XRN:5MWY46 | BRCA2 | SV | unknown | FALSE | 5699_6558>TA | S1901_S2186>L | 0.16 | chr13 | 32914192 | germline | not in tumor | Rev |
| prostate | XRN:WMDK15 | BRCA2 | SV | likely | FALSE | 2566_2835del270 | N856_K945del | 0.3909 | chr13 | | unknown | . | Rev |
| prostate | XRN:WMDK15 | BRCA2 | SV | likely | TRUE | 2779delA | M927fs*33 | 0.6352 | chr13 | 32911270 | unknown | homozygous | Sen |
| prostate | XRN:9F9N1D | BRCA2 | SV | likely | TRUE | 2809C>T | Q937* | 0.4889 | chr13 | 32911301 | somatic | homozygous | Sen |
| prostate | XRN:9F9N1D | BRCA2 | SV | likely | FALSE | 2779_2817del39 | M927_T939del | 0.5619 | chr13 | | unknown | . | Rev |
